# Alpha-ketoglutarate Potentiates IL-1β Production and Suppressive Mechanisms of Myeloid-Derived Suppressor Cells by Altering Redox Metabolism and Inducing Autophagy

**DOI:** 10.7150/ijbs.127309

**Published:** 2026-05-11

**Authors:** Marijana Milanović, Luka Pavlović, Marina Bekić, Jelena Đokić, Marija Stojadinović, Dušan Radojević, Miodrag Čolić, Sergej Tomić

**Affiliations:** 1 University of Defence ‒ Medical Faculty of the Military Medical Academy, Belgrade, Serbia.; 2 University of Belgrade ‒ Institute for the Application of Nuclear Energy, Department for Immunology and Immunoparasitology, Belgrade, Serbia.; 3 University of Belgrade ‒ Institute for Molecular Genetics and Genetic Engineering, Belgrade, Serbia.; 4 University of Belgrade ‒ Faculty of Chemistry, Centre of Excellence for Molecular Food Sciences, Department of Biochemistry, Belgrade, Serbia.; 5 Serbian Academy of Sciences and Arts, Belgrade, Serbia.; 6 University of East Sarajevo ‒ Medical Faculty Foča, Foča, Bosna and Hercegovina.

**Keywords:** alpha-ketoglutarate, myeloid-derived suppressor cells, tumour microenvironment, chronic inflammation, autophagy, immunotherapy

## Abstract

Alpha-ketoglutarate (αKG) has shown promise in cancer immunotherapy due to its profound impact on cancer cell metabolism and gene expression. However, its effects on myeloid-derived suppressor cells (MDSCs), critical immunosuppressive components of the tumour microenvironment, remain poorly understood. Using a model of GM-CSF/IL-6-induced monocyte-derived (mo)MDSCs we found that non-toxic doses of non-esterified αKG expanded CD14^+^HLA-DR^-/low^ moMDSCs, unlike esterified dimethyl-αKG. The αKG-moMDSCs displayed an enhanced suppressive phenotype, upregulated ILT-4 and IL-10, and showed an increased capacity to suppress Th1 cells. However, αKG-moMDSCs exhibited a heightened IL-1β production upon LPS stimulation, promoting Th17 cell expansion. This pro-inflammatory effect was consistent with oxoglutarate receptor (OXGR1)-dependent ROS increase, reduced Erk1/2 and Akt phosphorylation, reduced NRF-2 nuclear translocation and altered oxidative phosphorylation, as it could be mitigated by OXGR1-specific small interfering (si)RNAs delivered via lipid nanoparticles. Notably, the enhanced capacity of αKG-moMDSCs to suppress T cell proliferation and induce conventional FoxP3^+^ regulatory T cells was independent of OXGR1, relying instead on Atg5-mediated increase in autophagy flux and IL-10 production. Atg5 was dispensable for ILT-4 upregulation, but still essential for the induction of FoxP3^-^ type 1 and type 2 regulatory T (Tr1 and Tr2) cells, as well as OXGR1/ROS-mediated Th17 induction. Therefore, non-esterified αKG may promote moMDSC-mediated chronic inflammation and T cell dysregulation, potentially compromising its therapeutic efficacy in cancer immunotherapy.

## Introduction

Chronic inflammation is driven by a pathological immune response, which can lead to tumour progression, chronic infections and development of autoimmunity 1-3. Myeloid-derived suppressor cells (MDSCs), a heterogeneous population of immature, pathologically activated myeloid cells, are the hallmarks of chronic inflammatory conditions 4. These cells accumulate in the tumour microenvironment (TME), exerting profound immunosuppressive effects to facilitate tumour immune evasion 5,6. Monocytic (mo)MDSCs were shown to be more suppressive than polymorphonuclear (PMN)-MDSCs on a per-cell basis 7, due to their abundant immunosuppressive mechanisms. Namely, upon activation moMDSCs increase expression of metabolite-depleting enzymes (arginase (Arg)-1, indolamine-dioxygenase (IDO)-1, CD73, CD38), co-inhibitory receptors (program death ligand 1 (PD-L1), immunoglobulin-like transcript (ILT)3, ILT4, Notch-1) and immunosuppressive cytokines (interleukin (IL)-10, transforming-growth factor (TGF)-β, IL-27, etc.) 8,9 to efficiently suppress T cell response, both directly and indirectly via induction of regulatory T cells (Treg). However, moMDSCs were also demonstrated to produce proinflammatory mediators upon activation, i.e. IL-6 2, IL-1β 10 and polyamines 11. When combined with their immunosuppressive effects, this can lead to development of chronic inflammation. However, it is still poorly understood how different cues in tissue microenvironment, regulate these complex moMDSCs functions and deregulation of T cell response, yet a deeper understanding of these processes could drive the development of more effective immunotherapies for cancer and chronic inflammation.

Intermediates of the tricarboxylic acid (TCA) cycle emerged as critical factors governing immune cell functions 12,13. Thereby, alpha-ketoglutarate (2-oxoglutarate, αKG) metabolism is recognized as a key intermediate in TME connecting metabolic state to the epigenetic and functional programming of different myeloid cells 14. However, its effects and the mechanisms of actions in moMDSCs have not been studies previously. Previously, a-KG was shown to regulate cell growth and ageing via mammalian target of rapamycin (mTOR) and AMP-activated protein kinase (AMPK) 15, thereby significantly affecting the overall lifespan in several model organisms 16,17. Besides, αKG was shown to regulate hypoxia-inducible factor (HIF-1α) 18,19, epigenetic regulation of gene expression 20, as well as redox metabolism in various cell types 14. Interestingly, myeloid cells, but not lymphoid cells, were shown to express oxoglutarate receptor 1 (OXGR1, GPR99) 21 regulating the activation of downstream kinases such as phospho-inositol-3 kinase (PI3K), protein kinase B (Akt) and Extracellular signal-Regulated Kinase (Erk) 22. Previous papers indicated that HIF-1α-dependent mechanism induced by αKG in cancer cells could be beneficial for tumour therapy 23,24, and that αKG supplementation may potentiate the effects of checkpoint inhibitors (CKIs) 25. However, the pro-tumorigenic effects of αKG supplementation were demonstrated as well, particularly in immune cells 26,27. Namely, αKG was found to inhibit lipopolysaccharide (LPS)-induced differentiation of M1 macrophages and immunogenicity of DCs, leading to their tolerogenic and pro-tumorigenic functions 28,29. Therefore, it still remains unclear whether αKG is beneficial, or it might induce some severe adverse immunological effects in tumour therapy. Previous studies predominantly used esterified forms of αKG to assess its immunological effects 28,30,31. However, αKG analogues were demonstrated to rapidly hydrolyse extracellularly, and can induce analogue-dependent effects on cellular metabolism 32. Therefore, the aim of the current study was to investigate the effects of αKG on moMDSCs differentiation and suppressive functions in co-cultures with T cells, using a previously developed model of *in vitro* generated moMDSCs 9. Thereby, we hypothesized that distinct forms of αKG may exert different effects in moMDSCs, and that different actions of αKG on moMDSCs are mediated through OXGR1 and in OXGR1-independent manner.

## Materials and Methods

### Cells

The cytotoxicity and immunomodulatory properties of α-Ketoglutaric acid disodium salt dihydrate (αKG) and dimethyl-αKG (D-αKG) (both from Millipore Sigma, Taufkirchen, Germany) were tested *in vitro* on human peripheral blood mononuclear cells (PBMCs), and cultures of moMDSCs. Peripheral blood was collected from healthy donors, who provided written Informed consent before blood sampling, and all experiments were carried out in accordance with the Declaration of Helsinki after the study approval by the Ethical Committee of the Institute for the Application of Nuclear Energy (INEP). PBMCs were isolated from Na-EDTA-filled vacutainer tubes (Beckton Dickenson, New Jersey, USA) by density gradient centrifugation (Axis-Shield PoC AS, Oslo, Norway). Monocytes and total T cells, were purified from PBMCs by magnetic-activated cell sorting (MACS) using CD14-microbeads and MACS Pan T cell Isolation Kit, respectively (all from Miltenyi Biotec, Bergisch Gladbach, Germany), according to manufacturer's instructions, thus providing > 92% of purified CD14^+^ monocytes, and > 95% of T cells, as detected by flow cytometry (BD LSR II, Beckton Dickenson, California, United States).

### Experimental design

#### Peripheral blood mononuclear cells

Freshly isolated PBMCs (3x10^5^/well of the 96-wells plate) were cultivated in a complete RPMI-1640 medium with L-glutamine (2mM, Merk Sigma) or without L-glutamine, with 10% foetal calf serum (FCS), 50 µM 2-mercaptoethanol (all from Sigma-Aldrich, St. Louis, Missouri, USA), and antibiotics (Penicillin and Streptomycin, 100 U/ml, Merk Sigma). αKG stock solution (1 M) and D-αKG stock solutions (100 mM) were prepared fresh in RPMI medium without L-glutamine before each experiment, and the pH was set to 7.3 using NaOH and HCl when needed. PBMCs were treated with different doses of αKG (2.3 mM-150 mM) or D-αKG (0.62 mM-40 mM) and incubated for the next 72 h at 37 °C, 5% CO_2_ and 90% humidity, in the presence of phytohemagglutinin (PHA, Merk Sigma, 15 µg/ml) or its absence, followed by measurements of metabolic activity, proliferation and immunophenotype by flow cytometry.

The metabolic activity of PBMCs cultivated with different doses of αKG or D-αKG was analysed after 72 h in MTT assay. Blank controls included the same doses of αKG or D-αKG in complete medium but without cells. MTT (3-(4,5-dimethylthiazol-2-yl)-2,5-diphenyltetrazolium bromide, Merk Sigma) at the final concentration of 0.5 mg/mL, was added to each well for the next 4h. The cultures were then treated with 10% (w/v) sodium dodecyl sulphate (SDS, Millipore, Burlington, Massachusetts, United States) and 0.01N (v/v) hydrochloric acid (HCl, Merk Sigma) overnight to dissolve Formazan crystals. The absorbance was measured on a microplate reader at 570 nm (ELx800, BioTek, Winooski, Vermont, United States), and the reference wavelength was 670 nm, so the corrected OD = OD570 - OD670. The relative metabolic activity (MTT %) was calculated by subtracting the corresponding blank controls from the corrected OD values and normalizing the absorbance in control (non-treated) PBMCs to 100%.

The proliferation of PBMCs was determined by labelling the cells with Cell Trace Far Red (Invitrogen, CTFR 2.5 μM) and cultivating them with different doses αKG or D-αKG for 72h in the presence of PHA, followed by staining the cells with PI, and analysing CFTR dilution by flow cytometry (BD LSR II).

#### Monocytic myeloid-derived suppressor cells

To generate moMDSCs, MACS-purified monocytes were cultivated in complete RPMI medium supplemented at 2x10^6^ / 2mL/ well of 6 wells plate, in the presence of 20 ng/ml of human recombinant granulocyte macrophages colony-stimulating factor (GM-CSF; Novartis, Basel, Switzerland) and 20 ng/ml of human recombinant IL-6 (Bio-techne, RnD systems, Newcastle, UK) for 4 days 9; either in the presence of αKG (5mM or 30 mM), D-αKG (5mM) or their absence (control moMDSCs), starting from day 0. One-half of the medium containing the equivalent amounts of GM-CSF, IL-6 and αKG/D-αKG, was refreshed on day 3. In some experiments, the cells were treated with lipid nanoparticles (LNPs) encapsulating silencer siRNAs (1 µg/ml, as described below) at day -1, for the next 24 h. The cells were then washed in PBS and transferred to GM-CSF/IL-6 containing medium and cultivated in the presence or absence of αKG (30 mM) for the indicated period of time. The stimulation of moMDSCs was induced at day 4 of cultures with 100 ng/ml of LPS (Escherichia coli 0.111:B4, Merk Sigma) for next 16-18h. After the cultures, moMDSCs were harvested by light pipetting and washed twice in PBS, to remove the excess of free stimuli. The number of cells and viability were determined by using Muse cell count viability kit (Cytek Biosciences, Fremont, California, USA), and the analyses of cells' phenotype, proteins and genes expression, ROS, autophagy, OXPHOS and glycolysis were determined as described below, whereas the functions of moMDSCs were analysed in co-cultures with MACS-purified allogeneic T cells. Cell-free supernatants were collected and stored at -20 °C to detect cytokine levels.

#### Preparation and characterisation of lipid nanoparticles/siRNA

To determine the role of OXGR1 and autophagy related gene (ATG)5, in some experiments, the isolated monocytes were treated with LNPs containing silencer siRNAs targeting OXGR1 (Gene ID: 27199, s25983), ATG5 (Gene ID:9474), or scrambled FAM control (AM4620) (all from ThermoFisher Scientific). Silencer siRNAs were first encapsulated into LNPs using GenVoy-ILM T cell kit for mRNA (Cytiva, Marlborough, MA, USA). Briefly, concentrated GenVoy-ILM T cell lipid mix was placed into the smallest chamber of a microfluidic cartridge; twice the volume of liquid phase (22.5 µM siRNAs (≈0.3 mg/ml) in 1x Formulation buffer) was placed in the middle chamber; 3x volume was placed in the Dilution buffer recipient chamber. The single-use cartridges were placed into a purged Spark (Cytiva) at the formulation speed set to 3 or 4, depending on the initial volumes. The LNPs preparations were collected and diluted to the final concentrations of siRNAs at 75 µg/ml. The encapsulation efficiencies and yields were determined with Invitrogen Quant-it RiboGreen assay (ThermoFisher Scientific) according to manufacturer's instructions. LNPs size and number/concentration were determined by nanoparticle tracking analysis (NTA) on ZetaView (Particle Metrix), and by using Videodrop system (Myriade, Paris, France). The moMDSCs cultures were treated with freshly prepared LNPs/siRNAs at 1 µg/ml of encapsulated siRNA, along with recombinant human ApoE (Cytiva) (0.5 µg/ml) added immediately after the LNP/siRNAs, to facilitate LNPs internalization. The internalization of LNPs was assessed by treating the monocytes with LNP / fluorescently labelled FAM negative control siRNAs for 24h, followed by analysis on flow cytometer.

#### Mixed leukocyte reaction

The suppressive capacity and Th polarization potential of moMDSCs was tested in co-cultures with allogeneic T cells stimulated with anti-CD3/anti-CD28-coated Dynabeads (Thermo Scientific) and human recombinant IL-2 (10ng/ml RnD Systems). MoMDSCs (5x10^4^ - 0.625x10^4^/ well of the round-bottom 96-wells plate) were first washed twice in PBS to prevent any stimuli carryover, and then co-cultivated with CTFR-labelled T cells (1x10^5^/well), at 1:2 -1:16 moMDSC: T cell ratios, in the presence of Dynabeads (1:4, beads: T cell ratio) and recombinant IL-2 (10ng/ml, RnD Systems) for 4 days. After that, CTFR dilution was analysed by flow cytometry, after the excluding doublets and dead (propidium iodide (PI) +) cells. For the Th cell polarization assay, the co-cultures with moMDSCs were carried out in 1:4 moMDSC: T cell ratio for 5 days and then treated with Phorbol 12-myristate 13-acetate (PMA, 20 ng/mL) and ionomycin (500 ng/mL) (both from Merk Sigma) for the last 6 h, before harvesting the cell-free supernatants for cytokines quantification. To detect intracellular cytokines in T cells, the co-cultures were treated with PMA/ionomycin and monensin (2 μM, Sigma-Aldrich) for the last 4 h and then prepared for flow cytometry analysis. For the analysis of regulatory T cells, moMDSCs/T cell co-cultures were carried out in the presence of a low dose of human recombinant IL-2 (2 ng/ml) for 6 days, to facilitate Treg induction. In some experiments, the moMDSCs/T cell co-cultures were supplemented with blocking anti-ILT-4 Ab (2 μg/mL; R&D Systems), anti-IL-10 Ab (2 μg/mL, Bio-Rad Laboratories, Feldkirch, Germany), Ustekinumab-Stelara (5 μg/mL, Janssen Biotech, Inc., Horsham, PA, USA) or isotype control Ab (2 μg/mL anti-rat IgG2b; Thermo Fisher Scientific) to assess the contribution of ILT-4, IL-10, and IL-23, respectively, in these assays.

### Cell signalling

Phospho-ERK1/2 and phospho-Akt were determined in moMDSCs by using PI3K/MAPK Dual Pathway Activation Kit on Muse® Cell Analyzer (Cytek Biosciences, Fremont, CA, USA). Briefly, the cells were treated on day 0 with αKG (30 mM) and collected after 5 min, 10 min, 30 min, 4 h, 24 h or 48 h after the treatment, washed in PBS, fixed and permeabilized on ice for 10 min, and then stained with phycoerythrin (PE)-Cy5 labelled anti-phospho-ERK1/2 (Thr202/Tyr204, Thr185/Tyr187) and Alexa Fluor 555 anti-phospho Akt (Ser473), according to manufacturer's protocol. Reactive oxygen species (ROS) in moMDSCs were measured 48h or 72h after the treatment with αKG (30 mM) by using Muse Oxidative Stress Kit (Cytek Biosciences) containing dihydroethidium (DHE) dye as a probe. The autophagy flux was analysed in moMDSCs 48h after the treatment with αKG (30 mM), by using the LC3 Autophagy Kit (Cytek Biosciences), which is based on the detection of a membrane-converted variant of LC3 (LC3II). Briefly, the cells were washed first in PBS and then incubated in PBS in the presence of Bafilomycin, or in complete culture medium, for 4 h according to the manufacturer's protocol. The total expression of LC3-II was determined, and the autophagy flux was calculated as the ratio of bafilomycin-treated and non-treated cells in each experimental group.

### Oxygen consumption and extracellular acidification rates

Oxygen consumption rate (OCR, O2 mpH/ min), as a measure of oxidative phosphorylation (OXPHOS), and extracellular acidification rate (ECAR, mpH/ h), as a measure of glycolysis, were determined in moMDSCs collected after the differentiation and stimulation with LPS, by using MitoXpress Xtra and pH-Xtra kits (both from Agilent Technologies, Santa Clara, CA, USA), respectively, as described previously 29. Briefly, moMDSCs were washed in PBS, seeded at 7.5 x 10^4^ cells/well Black Polystyrene Plates RPMI containing 25 mM glucose, 1 mM pyruvate and 2 mM L-glutamine, and the OCR was measured at basal rate or in cells treated with 2 μM oligomycin, 0.5 μM FCCP [0.5 mM carbonyl cyanide 4-(trifluoromethoxy) phenylhydrazone] or 1 μM Rotenone/ 1 μM Antimycin A (all from Merk Sigma). OCR dye and HS Mineral Oil were added to each well and the readings were made in dual-read TRF mode (Victor3V, Perkin Elmer Inc., Waltham, MA, USA) at ~1 min for total of 60 minutes. For ECAR measurements, moMDSCs were cultivated in an unbuffered DMEM (wo bicarbonate, with 2 mM L-glutamine and 143 mM NaCl) at the basal rate, or after the treatment with 2 μM oligomycin or 20 mM 2-Deoxyglucose (2-DG). The lifetime fluorescence for OCR and pH for ECAR were calculated in Data Visualization Tool V 1.27 (Agilent Technologies) and Graph Prism 8.0 software.

### Western blot

MoMDSCs were harvested at 48h or 5 days after the treatment with αKG. The cells were lysed in RIPA buffer (50 mM Tris-HCl pH 7.4, 150 mM NaCl, 0.25% sodium deoxycholate, 1 mM EDTA, 1% Triton X-100, 0.1% SDS) supplemented with protease (cOmplete™, Roche, Basel, Switzerland) and phosphatase inhibitors (PhosSTOP™, Roche), for 25 min on ice, centrifuged at 13,000×g for 20 min, and the protein concentration in lysate was determined using the Pierce™ BCA Protein Assay Kit (Thermo Fisher Scientific, Waltham, MA, USA). The protein (15 μg)/sample were resolved on 12.5% SDS-PAGE gels and transferred to 0.45 μm PVDF membranes (Immobilon®-P, Millipore, Billerica, MA, USA) using a Mini Trans-Blot system (Bio-Rad, Hercules, CA, USA). Membranes were blocked in 5% non-fat dry milk in TBS-T (20 mM Tris-HCl, 150 mM NaCl, 0.1% Tween 20, pH 7.4) for 1.5 h at room temperature, then incubated overnight at 4 °C with primary polyclonal Abs: anti-HO-1, anti-Keap1, anti-LC3B, anti-NRF2, anti-β-actin (all at 1:1000 dilution), and GAPDH (1:3000 dilution; all obtained Thermo Fisher Scientific), and monoclonal anti-ATG5 Ab (MAB5294, 1:750; R&D Systems, Minneapolis, MN, USA). Secondary HRP-conjugated goat anti-rabbit Ab (1:10,000 dilution; Thermo Fisher Scientific) or goat anti-mouse (1:1000; R&D Systems) Abs were used for visualisation after using Pierce™ ECL substrate (Thermo Fisher Scientific) on a ChemiDoc™ MP Imaging System (Bio-Rad) and quantified with ImageJ software (v1.54g, NIH, Bethesda, MD, USA). Target proteins were normalized to corresponding loading controls (GAPDH or β-actin) as indicated (Additional File 1).

### Epifluorescent microscopy

Cytospins were prepared from moMDSCs collected during their differentiation from monocytes on Suprefrost slides, using the Rotofix 32A centrifuge with Cytorotor 1515-A (Hettich Centrifuge, Tuttlingen, Germany) and placing 2x10^4^/ 50µl PBS at 500 RPM for 5 minutes. OXGR1 expression was analysed on samples cultivated for 30 min, 2h, 48h or 5 days, followed by staining of air dried slides with recombinant rabbit-OXGR1 Ab at 1:100 dilution (bsm-62646r, Bioss Inc., Woburn MA, USA) overnight at +4 °C, followed by secondary anti-rabbit Alexa 488 Ab at 1:1000 dilution for 1h at room temperature, and DAPI (4′,6-diamidino-2-phenylindole) for 30 minutes (both from Thermo Fisher Scientific). The samples of moMDSCs cultivated for 5 days were also stained with anti-NRF2-Alexa fluor 488, anti-ILT4-PE (all from Biolegend) and HIF-1α:biotin (H1alpha67) (Thermo Fisher Scientific) in humid chamber at 4 °C overnight. After washing in PBS, they were incubated with Streptavidin Alexa Fluor™ 647 and DAPI (both from Thermo Fisher Scientific) for 30 minutes at room temperature. The slides were mounted in mounting medium (Millipore Sigma) and analysed on Zeiss AxioImager A1 under a UV filter set for DAPI (UV-2B, ex: 330-380 nm, DM 400, BA 435), a green filter set for detection of OXGR1 or NRF2 (B-2A, ex: 450-490 nm, DM 505, BA 520), red filter set for detection of ILT4 (G-2A, ex: 510-560 nm, DM 575, BA 590) and far-red filter set 50 for detection of HIF-1α (ex-620-650 nm, DM 660 nm, BA670). The images for epifluorescent microscopy were acquired as monochromatic and analysed offline using the ImageJ software (National Institutes of Health, Bethesda, Maryland, USA).

### Quantitative polymerase chain reaction

Total RNA was extracted with Trizol reagent (Invitrogen) from moMDSC collected 48h after the treatment with αKG, and treated with DNase I using RapidOut DNA Removal Kit (Thermo Fisher Scientific) to remove genomic DNA. RevertAid RT kit, Random hexamers, and RiboLock RNase inhibitor (all from Thermo Fisher Scientific) were used for reverse transcription of 100 ng of isolated RNA from each sample. Synthesized cDNA was amplified in Line-Gene 9600 Plus Real-Time PCR (Hangzhou Bioer Technology) by using IC Green qPCR Universal Kit (NIPPON Genetics, Düren, Germany) under the following conditions: 2 min at 95 °C activation, 40 cycles of 5 s at 95 °C and 30 s at 60 °C. Primers used for qPCR are shown in Supplementary Table S1. The results were normalized against the glyceraldehyde-3-phosphate dehydrogenase (GAPDH) and expressed as relative target abundance using the 2^-ΔΔCt^ method.

### Flow cytometry

Flow cytometry was used to assess the phenotype of PBMCs, moMDSCs and T cells after the cultures. The cells were washed once in PBS/ 2% FCS/ 0.01% Na-azide (Sigma-Aldrich) and incubated with Human TrueStain FcX (Biolegend) for 15 minutes prior to labelling them with fluorochrome-conjugated monoclonal antibodies (clones) at the dilutions recommended by the manufacturer. Antibodies used for flow cytometry and the staining protocol are provided in Supplementary Information.

### Cytokine measurements

The supernatants moMDSCs cultures were collected after 5 days cultivation and assayed for cytokine measurements using sandwich enzyme-linked immunosorbent assay (ELISA) for IL-12p70, IL1b, IL-10, IL-23, TNF-α, IL-27 and TGF-β (all from R&D Systems), followed by normalization of cytokine levels to the same viable number of cells. LEGENDplex™ Th-plex panel, detecting IL-10, IL-9, IL-22, IL-17F, IL-17A, IL-13, IL-5, IL-4, IL-6, TNF-α and IFNγ, was used to measure cytokines produced in moMDSC/T cell co-cultures. All cytokine measurements were done in duplicates, and the concentrations were calculated according to 5-parameter nonlinear fit curves (GraphPad Prism 8).

### Statistical Analysis

To analyse the differences between the αKG-treated and control MDSCs, a repeated-measures one-way or two-way analysis of variance (RM-ANOVA) with Geisser-Greenhouse's correction for sphericity were performed, followed by Dunnett's or Tukey's multiple comparison test, respectively (GraphPad Prism 8). Data are presented as means ± SEM of the indicated number of independent experiments and a 95% confidence interval was taken as a significant difference between the tested groups. The heat map for cytokine concentrations was prepared by ranging the values from each experiment from 0-1 according to the formula: range value = (value - minimal value) / (maximal value - minimal value).

## Results

### Non-esterified αKG potentiates differentiation of monocyte-derived MDSCs which produce IL-10 and IL-1β

**Figure 1:**
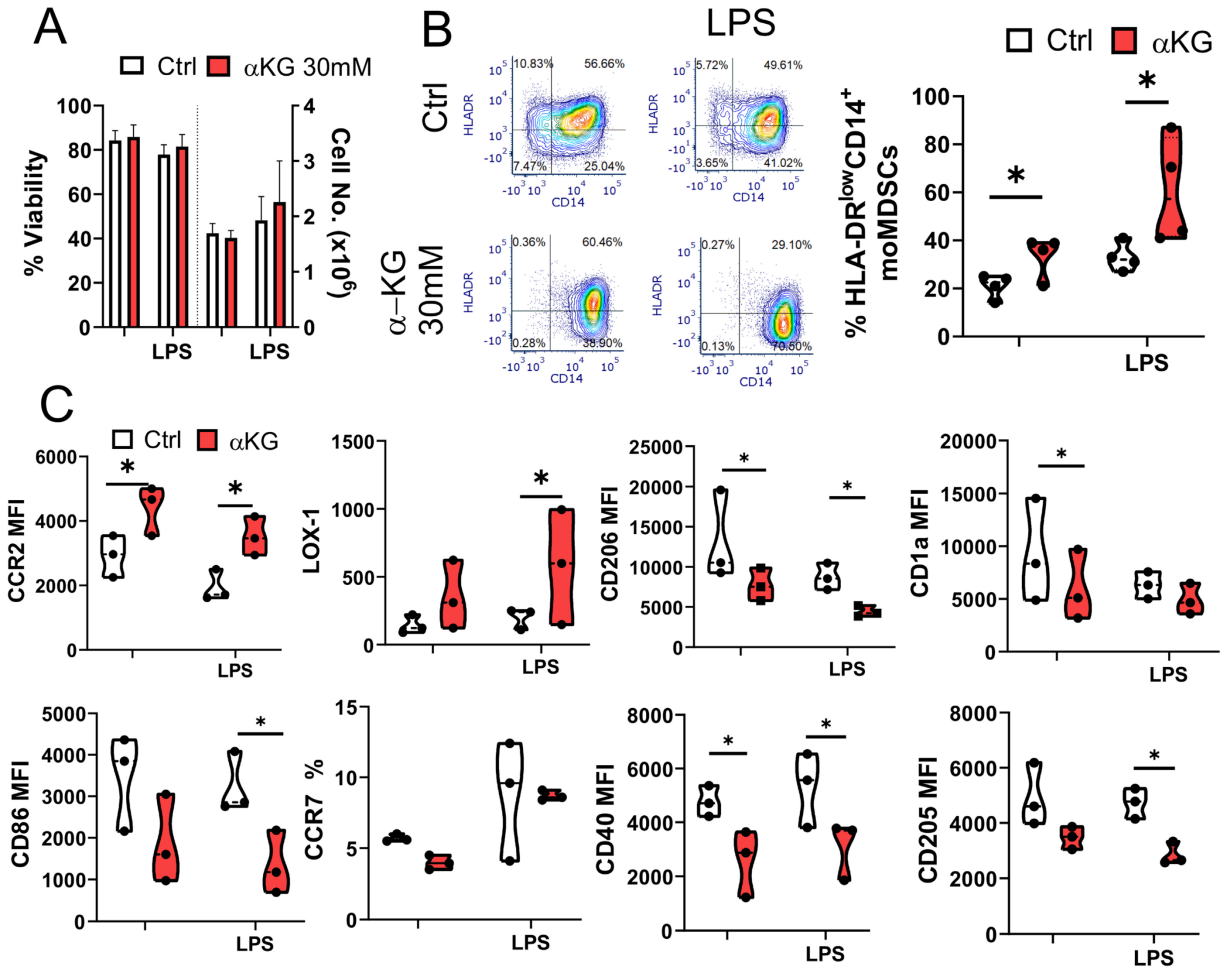
** Phenotype of moMDSCs differentiated with αKG.** CD14^+^ monocytes were differentiated into moMDSCs for 5 days with GM-CSF and IL-6, with or without 30 mM αKG from day 0, and with or without LPS for the last 16-18 h. **A)** Viability assessed by PI/acridine orange staining. **B)** Representative flow cytometry data for HLA-DR and CD14 expression, with summarized % of HLA-DR^low/-^CD14^+^ moMDSCs. **C)** Expression of indicated markers analysed by flow cytometry, measuring mean fluorescence intensity (MFI) or % of positive cells. **(A-C)** Data are presented as violin plots with median and quartiles, with dots of independent experiments. *p < 0.05, as indicated (RM-ANOVA, Dunnett's post-test).

MoMDSCs were generated from purified CD14^+^ monocytes in the presence of GM-CSF and IL-6 for 4 days 9, either in the presence of αKG or its absence, followed by 16-18 h stimulation with LPS, a ligand for TLR4 which was demonstrated as highly relevant for myeloid cells in TME 33, and one of the key activators of MDSCs in a two-step activation process 4. The selected doses of non-esterified αKG (30 mM), commonly used in *in vitro* studies 34,35 including our own 29, were stable in cell-free medium (Supplementary Figure S1). The applied dose of αKG displayed no toxicity in moMDSCs cultures (Figure 1A), and similar data was obtained in PBMCs cultures (Supplementary Figure S2A), used as an additional model system. The moMDSCs differentiated with αKG displayed significantly reduced HLA-DR and increased CD14 expression compared to control moMDSCs, indicating an overall increase in the proportion and number of CD14^+^HLA-DR^low^ moMDSCs in those cultures. Down-regulation of HLA-DR was even more potentiated in αKG-treated cells stimulated with LPS (Figure 1B). Additionally, the activity of cathepsin V, an enzyme involved in antigen processing for HLA-DR presentation 36, was also found reduced in αKG-treated vs control moMDSCs after differentiation (Supplementary Figure S3). Moreover, we observed an increased expression of CCR2 and LOX-1 by αKG-treated moMDSCs, irrespective of additional LPS stimulation. αKG-treated moMDSCs showed reduced expression of DC/macrophage markers (CD206, CD205, CD1a), and costimulatory molecules (CD86 and CD40) (Figure 1C), indicating an enhanced immunosuppressive phenotype 37,38. Similar effects of non-esterified αKG on moMDSCs phenotype were observed in culture media with no L-glutamine, as well as in media supplemented with L-glutamine (Supplementary Figure S4), suggesting that the effect was not related to L-glutamine supplementation. Additionally, a prolonged differentiation of moMDSCs in GM-CSF / IL-6 for 7 days, instead of 4 days, did not diminish the effects of αKG on downregulation of HLA-DR and CD1a, and the upregulation of CD14 and CCR2 (Supplementary Figure S5). Interestingly, the effects of non-esterified αKG on HLA-DR expression were opposite to those induced by the esterified D-αKG form (5 mM), whereas the changes in expression of CD14, CCR2, LOX1 and CD86 were more pronounced with the non-esterified αKG (Supplementary Figure S4), suggesting that non-esterified αKG and D-αKG might have different immunomodulatory effects.

To test this, we compared the effects of αKG and D-αKG in PHA-stimulated PBMC cultures, as a more robust model system of immune response. Thereby, the non-toxic dose of non-esterified αKG displayed anti-proliferative effects (Supplementary Figure 2B), and it lacked the capacity to activate CD4^+^ and CD8^+^ T cells (Supplementary Figure 2C, D). In contrast, esterified D-αKG, which displayed higher toxicity (toxic at doses ≥ 10mM, Supplementary Figure S2A), did not suppress the proliferation of PHA-PBMCs in non-toxic doses, and it triggered the activation of T cells, according to increased expression of HLA-DR/CD38 and CD25/CD69 on these cells (Supplementary Figure S2B, C, D). These results suggested that the non-esterified αKG displays potent immunoregulatory effects in human moMDSCs and PHA-PBMCs, and that these effects are different from those induced by D-αKG. Considering that esterified forms of αKG can induce αKG-independent, ester-groups-dependent metabolic changes in target cells 32, we focused further study on non-esterified form of αKG.

**Figure 2:**
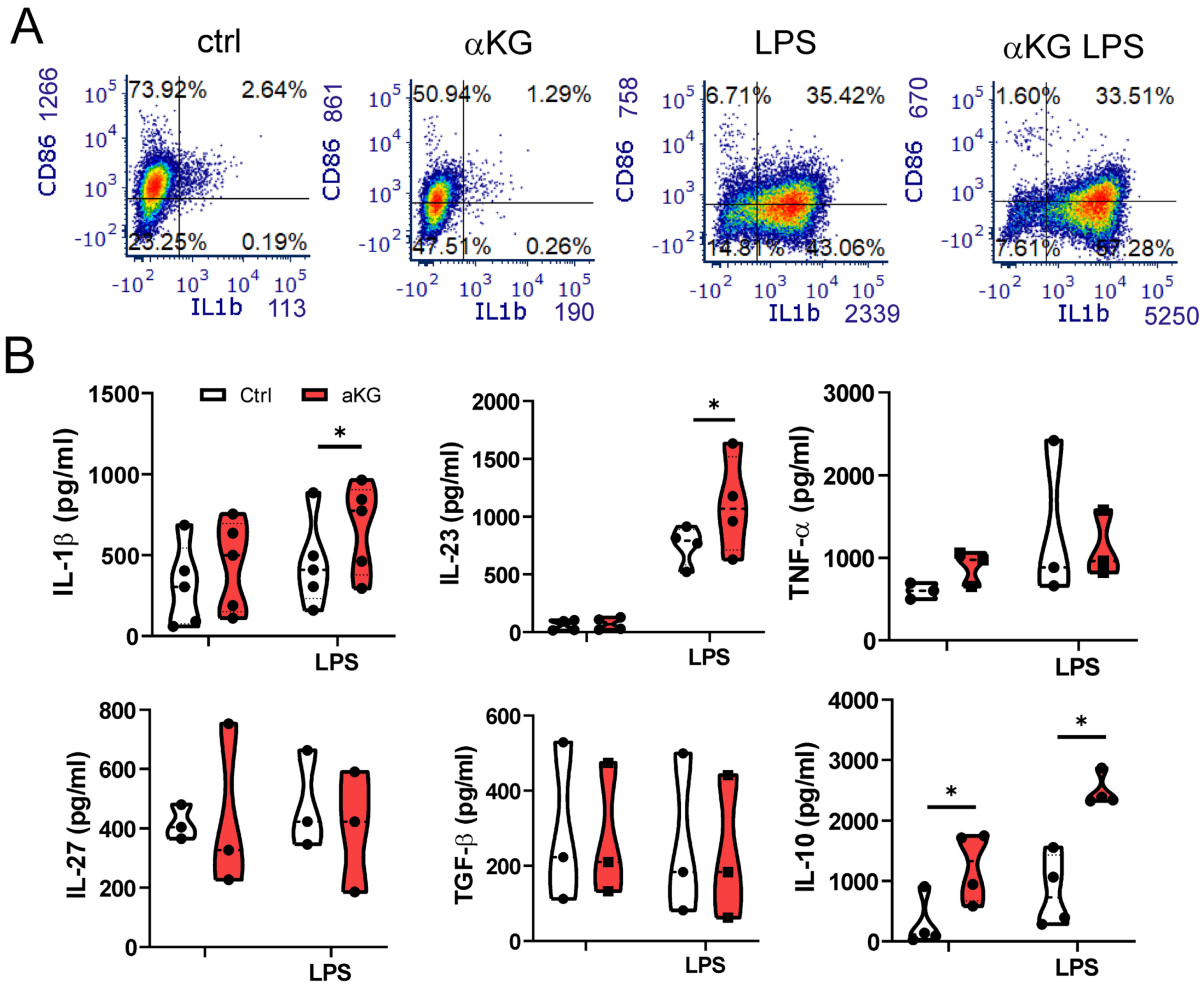
**Cytokines production by moMDSCs differentiated with αKG.** moMDSCs were differentiated for 5 days with GM-CSF and IL-6, with or without 30 mM αKG from day 0, and with or without LPS for the last 16-18 h. **A)** Representative flow cytometry data for CD86 and IL-1β expression out of 4 experiments with similar data. **B)** Levels of cytokines measured by ELISA from culture supernatants, and normalized to 1x10^6^ viable cells. **(A-B)** The data are shown as violin plots with median and quartiles, with dots of independent experiments. *p < 0.05, as indicated (RM-ANOVA, Dunnett's post-test).

When analysing cytokines produced by moMDSCs, we found that αKG significantly potentiated the capacity of moMDSCs to produce immunoregulatory cytokine IL-10, both in non-stimulated and LPS stimulated cultures (Figure 2). However, αKG-treated moMDSCs also produced an increased levels of pro-inflammatory cytokine IL-1β, and its direct responder IL-23 involved in Th17 polarization 39, upon stimulation with LPS (Figure 2A, B). In contrast, the production of TNF-α, IL-27 and TGF-β were not affected significantly, whereas the production of Th1-polarizing cytokine IL-12p70 was below the detection limit in moMDSCs cultures.

### αKG alters oxidative metabolism in moMDSCs via oxoglutarate receptor and reduced phosphorylation of Erk1/2 and Akt

**Figure 3:**
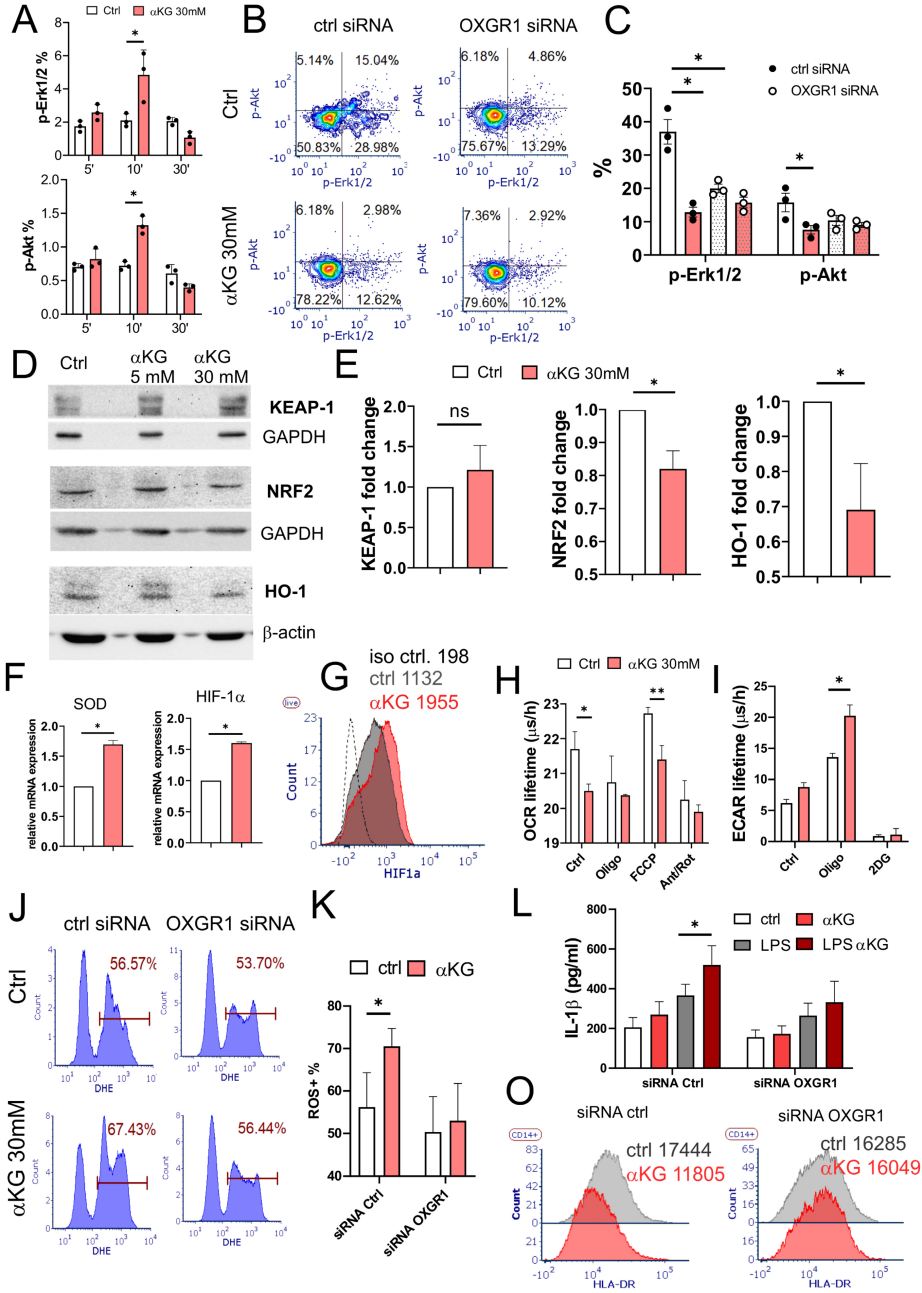
** Role of OXGR1 in response of moMDSCs to αKG. A)** CD14^+^ monocytes were treated with αKG (30 mM) in PBS for 5, 10 or 30 minutes, followed by analysis of pErk1/2 and pAkt upon staining the cells with PI3K/MAPK Dual Pathway Activation Kit on Muse® Cell Analyzer (see also Supplementary Figure S6 A,B for 4h, 24h and 48h data). **B)** Representative analysis of pErk1/2 and pAkt in cells treated with 1 µg/mL OXGR1-siRNA or scrambled (ctrl) siRNA encapsulated in LNPs with 0.5 µg/mL ApoE (Supplement Figure S7) for 24 h, prior to differentiation with GM-CSF/IL-6, with or without 30 mM αKG, for the next 48 h, and **C)** the summarized data are shown. **D)** Representative western blots of Keap-1, Nrf-2 and HO-1, and loading controls (GAPDH or β-actin) from moMDSCs treated on not with αKG (5mM or 30 mM) for 48h, and **E)** the summarized data with 30 mM αKG, normalized to control moMDSCs. **F)** Data from qPCR of SOD1 and HIF-1α relative mRNA expression from cells treated as in (D) from one experiment in triplicates, out of two similar. **G)** Representative data on HIF-1α expression from moMDSCs treated or not with αKG for 5 days, out of four independent experiments. **H)** OXPHOS (OCR lifetime µs/h) and glycolysis (ECAR lifetime µs/h) measured in moMDSCs differentiated with or without αKG (30 mM) for 5 days, and treated with Oligomycin (Oligo, 2 μM), FCCP (0.5 μM), Anthocyanin/Rotenone (2 μM each), or 2-deoxy glucose (2DG, 20 mM) during the measurements (see Supplementary Figure S6D, E for representative kinetic measurements). **J)** Representative analysis of ROS in moMDSCs treated as in (B), after 5 days of cultures, and **K)** summarized data from 3 independent experiments. **L)** IL-1β concentrations measured from supernatants of moMDSCs treated as in (B) after 5 day cultures. **O)** Representative data on HLA-DR expression in moMDSCs prepared as in (B). **(A,C,E, H, K, L)** Data is shown as mean ± SEM. *p < 0.05, **p < 0.01 as indicated (RM-ANOVA, Dunnett's post-test).

The activation of OXGR1 was shown to induce transient phosphorylation of Erk1/2 40. Accordingly, the treatment of purified monocytes with αKG in PBS induced an increase in p-Erk1/2 and p-Akt compared to control cells, peaking at 10 min after the treatments (Figure 3A). However, the measurements of pErk1/2 and p-Akt at 4h, 24h and 48h cultures, revealed their reduced phosphorylation in αKG-treated cells compared to control (Supplementary Figure S6A, B). Thereby, intracellular Ca^2+^ levels were consistently increased in αKG-treated moMDSCs compared to control (Supplementary Figure S6C). To assess whether this phenomenon is OXGR1-mediated, the cells were pre-treated with LNPs carrying OXGR1-specific siRNAs (Supplementary Figure S7) for 24h, and then with αKG for the next 48h. OXGR1-silenced cells showed reduced p-Erk1/2 and p-Akt levels as compared to non-silenced cells, but αKG had no additional effects on p-Erk1/2 and p-Akt levels of OXGR1-silenced moMDSCs (Figure 3B, C). These results indicate that OXGR1 ligation by αKG or its silencing with siRNA induces a continuously reduced level of Erk1/2 and Akt phosphorylation during moMDSCs differentiation.

Nrf2, a key regulator of oxidative metabolism, is tightly regulated by Erk1/2 phosphorylation 41. In line with this, we observed a reduced expression of Nrf2 protein and its downstream target HO-1 by WB in moMDSCs treated with αKG, although KEAP-1 levels were insignificantly increased (Figure 3D, E). In contrast, we found increased mRNA expression of superoxide dismutase (SOD)1 and HIF-1α in αKG-treated moMDSCs, as compared to control moMDSCs (Figure 3F). Flow cytometry analysis confirmed that HIF-1α protein levels were indeed increased in αKG-treated cells compared to control cells (Figure 3G).

Since HIF-1α is a key metabolic switch regulating OXPHOS 42, we measured OCR and ECAR in moMDSCs and found that αKG-treated cells display reduced OXPHOS and increased glycolysis rate (Figure 3H, I, Supplement Figure S6D, E). In line with this, αKG-treated cells displayed increased ROS levels, but not when the cells were previously silenced for OXGR1 (Figure 3 J, K). Namely, OXGR1 silencing in moMDSCs was able to prevent αKG-induced ROS and IL-1β production upon LPS stimulation (Figure 3L). Additionally, OXGR1 silencing prevented αKG-induced down-regulation of HLA-DR in moMDSCs (Figure 3O). These results suggested that αKG induces dephosphorylation of Erk1/2 and Akt, and a metabolic shift towards increased glycolysis, HIF-1α stabilization, ROS-mediated inflammasome activation, and overall expansion of moMDSCs.

### αKG induces autophagy in moMDSCs independently of OXGR1

**Figure 4:**
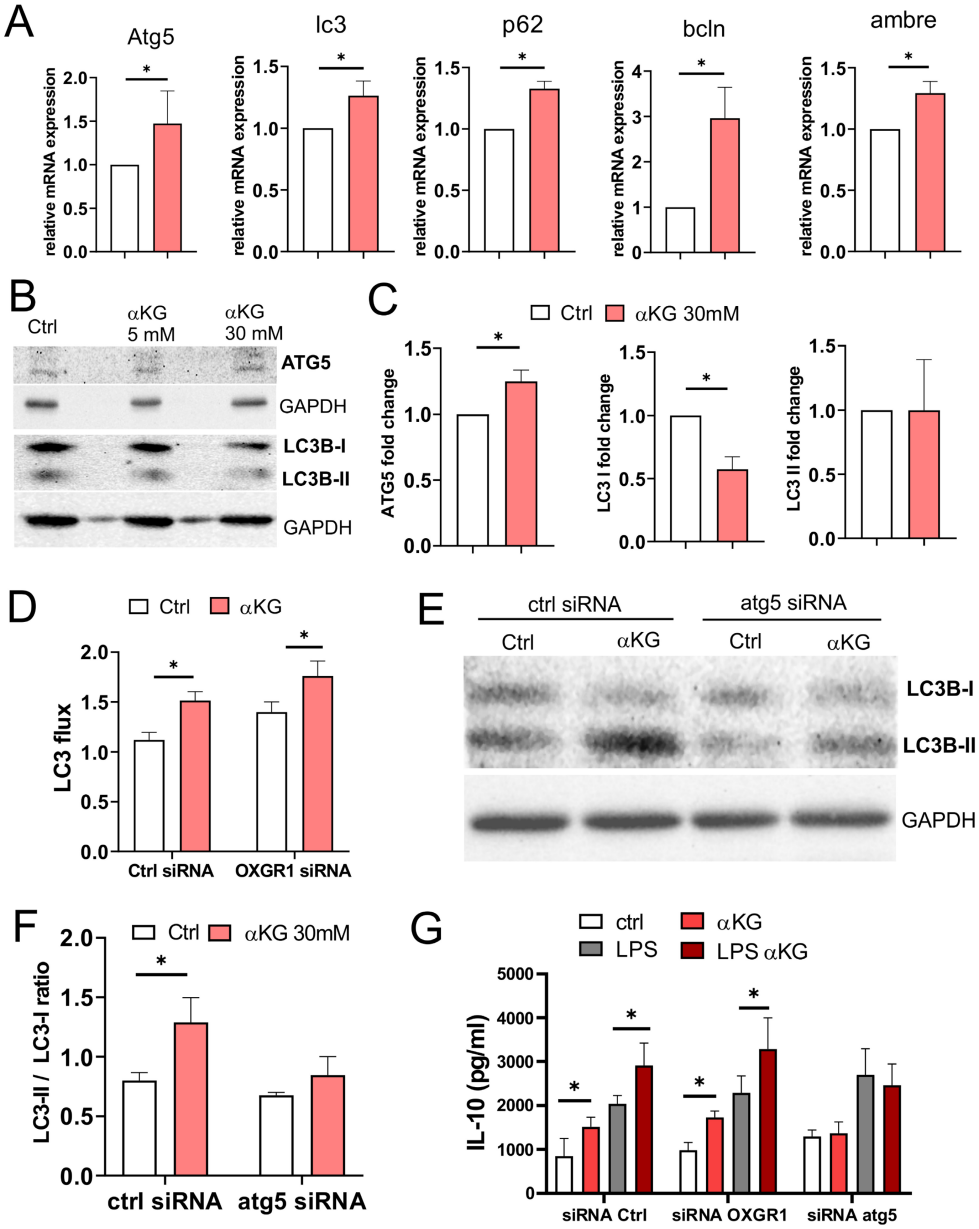
**Role of autophagy in response of moMDSCs to αKG. A)** Relative Atg5, lc3, p62, bcln and ambre mRNA expression, measured by qPCR from moMDSCs treated or not with αKG (30 mM) for 48h is shown from one experiment carried out in triplicates, out of 2 experiments with similar results. **B)** Representative wester blots of ATG5, LC3B-I, LC3B-II with GAPDH as loading control from moMDSCs treated on not with αKG (5mM or 30 mM) for 48h, and **C)** the summarized data normalized to control moMDSCs (1) are shown. **D)** MoMDSCs pretreated with 1 µg/mL siRNA^oxgr1^ or control scrambled siRNA (encapsulated in LNPs with 0.5 µg/mL ApoE, Supplement Figure S7) for 24 h, and then differentiated with GM-CSF and IL-6 with or without 30 mM αKG for 4 days were analysed for LC3 flux (ratio between bafilomycin-treated and non-treated cells) after staining for membrane-bond LC3 expression and the analysis on Cell Muse Analyser (see Supplementary Figure S8A for representative histograms). **E)** Representative western blot of LC3B-I and LC3B-II with GAPDH as loading control in moMDSCs pretreated with 1 µg/mL siRNA^atg5^ or control siRNA, as in D (see Supplementary Figure S8 for the equivalent LC3 flux experiment), and **F)** the summarized data are shown. **G)** IL-10 concentrations in culture supernatants of moMDSCs, pretreated with siRNAs (as in D and E), and then treated or not with αKG (30mM) for 5 days and LPS (100 ng/ml) for the last 16-18h, as indicated. **(C,D, F,G)** Data is shown as mean ± SEM of 3 independent experiments. *p < 0.05 as indicated (RM-ANOVA, Dunnett's post-test).

Oxidative metabolism, autophagy, and inflammation are mutually tightly regulated 43. We measured the expression of autophagy-related genes in moMDSCs and found that αKG-treated cells displayed increased mRNA levels of Atg5, Lc3, p62, beclin (bcln) and Ambre, compared to control cells (Figure 4A). WB analysis indicated an increased expression of Atg5 levels in cells treated with 30mM αKG, as well as increased LC3B-II/LC3B-I ratio, predominantly due to reduced LC3B-I expression (Figure 4B, C). These results suggested that αKG might increase autophagy flux in moMDSCs and protein turnover via lysosome degradation. Indeed, by blocking the lysosomal degradation with bafilomycin and measuring only the membrane-bound LC3-II fraction, we observed a direct increase in LC3-II expression and autophagy flux in αKG-treated moMDSCs compared to control moMDSCs (Figure 4D, Supplement Figure S8). Interestingly, the silencing of OXGR1 in moMDSCs did not reduce αKG-induced increase in autophagy flux, suggesting that this effect is OXGR1-independent. In contrast, silencing of Atg5 in moMDSCs, by using LNPs delivering Atg5-specific siRNAs (Supplementary Figure S7), prevented αKG-induced increase in LC3II/LC3I ratio (Figure 4E, F, Supplementary Figure S8). Furthermore, the capacity of αKG-treated moMDSCs to upregulate IL-10 production was impaired in cells silenced with Atg5-siRNAs, but not in cells silenced with OXGR1-siRNAs, suggesting that IL-10 upregulation in αKG-moMDSCs was dependent on autophagy induction, rather than on OXGR1 signalling (Figure 4G).

### αKG-treated moMDSCs suppress Th1, while promoting Th2 and Th17 polarization

**Figure 5:**
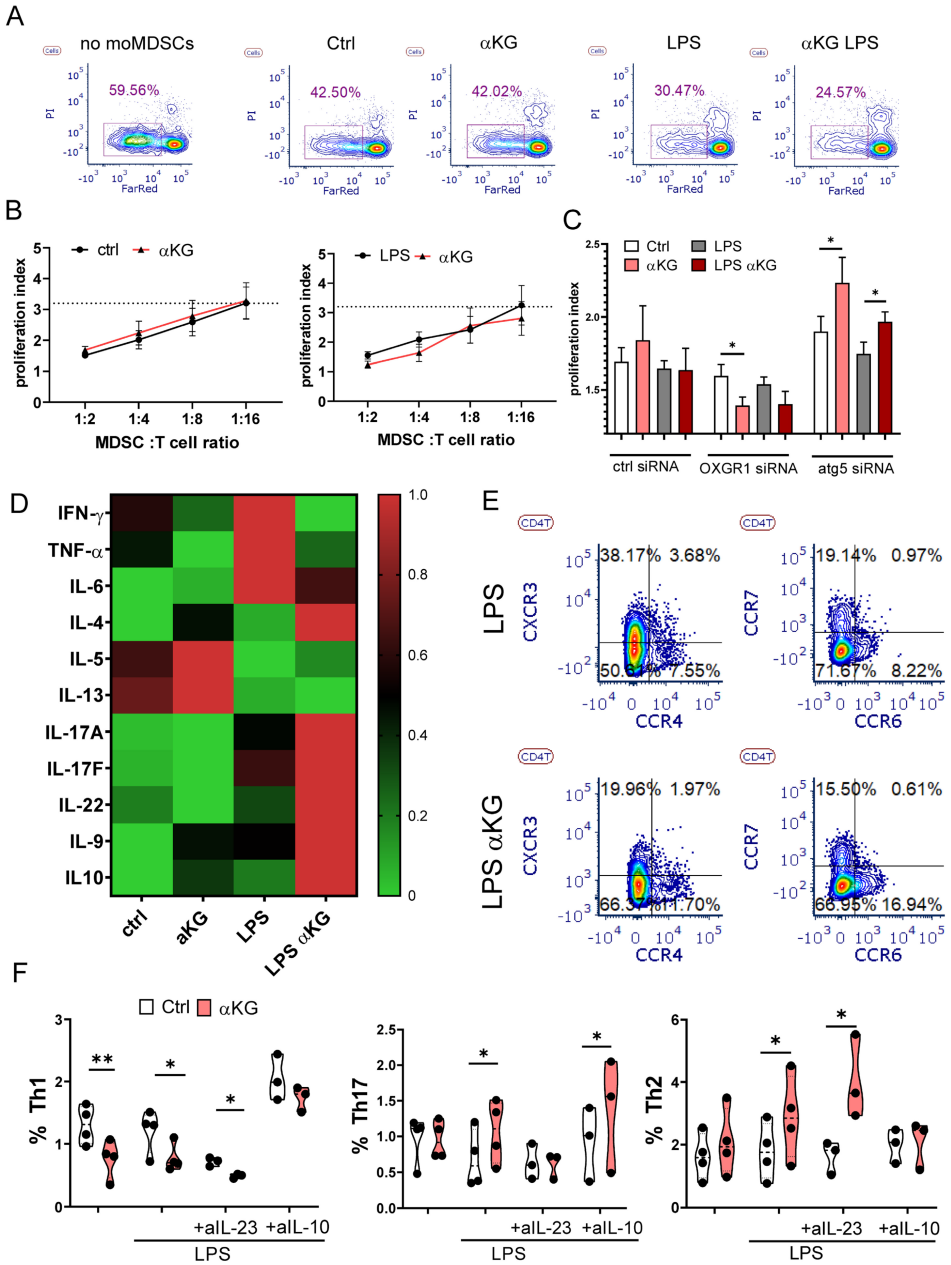
** Functional properties of αKG-moMDSCs in co-cultures with T cells.** MoMDSCs, generated with αKG (30 mM) or without it (ctrl), and stimulated or not with LPS (100 ng/ml), were co-cultivated with allogeneic T cells (1x10^5^/well) in the presence of CD3/CD28 Dynabeads and IL-2. **A)** A representative analysis of T cells proliferation, prelabelled with CTRF, from 4-day co-cultures with moMDSCs at 1:4 (moMDSC: T cell ratio), followed by staining of dead cells with PI, and **B)** the summarized data of proliferation index at different moMDSC: T cell ratios are shown with a dotted line, representing the average proliferation index of T cells cultivated without moMDSCs. **C)** Proliferation index of T cells co-cultured with moMDSCs that were pre-treated with OXGR1-siRNA, Atg5-siRNA or scrambled (ctrl) siRNA for 24h and then αKG and LPS, as described. (B, D) data is shown as mean proliferation index ± SEM (n=3). **D)** Heat-map representing relative cytokine levels supernatants collected from 5-day moMDSC/T cell co-cultures as in (A), and normalizing the average values (n=3) to 0-1 range. **E)** Representative contour-plots of CXCR3, CCR4, CCR6 and CCR7 expression in CD4^+^ T cells, from 5-day moMDSCs/T cell co-cultures as in (A). **F)** Summarized data on % of Th1 (IFN-γ^+^), Th17 (IL-17^+^) and Th2 (IL-4^+^) in CD4^+^ T cells (gated as in Supplement Figure S9) from 5-day co-cultures with moMDSCs as in (A) carried out in the presence or absence of blocking Abs Ustekinumab (anti-IL-23) or anti-IL-10 (both at 2 µg/ml) or irrelevant Ab, are shown as violin plots with median and quartiles, with dots of independent experiments. *p < 0.05 as indicated (RM-ANOVA, Dunnett's post-test).

To evaluate the effects of moMDSCs on T cell proliferation and polarization, we have co-cultivated allogeneic T cells stimulated with CD3/CD28-coated Dynabeads with or without moMDSCs. Expectedly, moMDSCs strongly suppressed proliferation of T cells, especially in higher moMDSCs: T cell ratios (Figure 5A, B). However, no significant differences were found between αKG-treated and control moMDSCs, irrespective of their treatment with LPS. Interestingly, when OXGR1 was silenced in moMDSCs, αKG-treated cells displayed an enhanced suppressive potential compared to corresponding control moMDSCs. In contrast, when Atg5 was silenced, αKG-moMDSCs displayed a significantly reduced suppressive potential (Figure 5C). These results suggested that the suppressive properties of moMDSCs are differently regulated by OXGR1 and Atg5, probably due to different subtypes of T cells being potentiated in the co-cultures.

To test this hypothesis, we analysed T cell phenotype and detected a reduced proportions of naïve (CD45RA^+^CCR7^+^) CD4^+^ and CD8^+^ T cells, and increased proportions of effector/memory T cells in co-cultures with αKG-moMDSCs (Supplementary Figure S9). The quantification of T cell-related cytokines from moMDSCs/T cell co-cultures showed that αKG-moMDSCs co-cultures contained reduced levels of Th1 cytokines (IFN-γ and TNF-α), and increased levels of Th2 (IL-4, IL-5, IL-13), Th9 (IL9), Th17 cytokines (IL17A, IL17F, IL-22), as well as increased levels of IL-10 (Figure 5D). Thereby, the effects of αKG-moMDSCs on IL-5 and IL-13 was more pronounced in co-cultures without LPS stimulation, while all other changes were most prominent in co-cultures with LPS-stimulated moMDSCs. In line with these results, we found reduced expression of CXCR3 (Th1-related) on Th cells, and increased expressions of CCR4 (Th2-related) and CCR6 (Th17-related) on Th cells co-cultured with αKG-moMDSCs, relative to control (Figure 5E). Finally, intracellular cytokine staining confirmed that the Th cells cultivated with αKG-moMDSCs contained lower levels of IFN-γ and TNF-α (i.e. lower % of Th1 cells) and higher levels of IL-17 and IL-4 compared to Th cells cultivated with control moMDSCs (Figure 5F, Supplementary Figure S9). The potentiation of Th17 cell differentiation was dependent on IL-23 production by LPS-stimulated αKG-moMDSCs, as it could be blocked by Ustekinumab during the co-cultures (Figure 5F). The suppression of Th1 cell differentiation and expansion of Th2 cells in co-cultures with αKG-moMDSCs was mostly depended on IL-10, as it could be blocked by anti-IL-10 Abs (Figure 5F). Similar effects of αKG-moMDSCs in co-cultures were observed when analysing the expression of IL-17 and IL-4 in CD8^+^T cells, but the production of IFN-γ and TNF-α by these cells was not modified significantly, particularly after LPS stimulation (Supplementary Figure S9).

### αKG-induced autophagy in moMDSCs is necessary for the induction of regulatory T cell subsets

**Figure 6:**
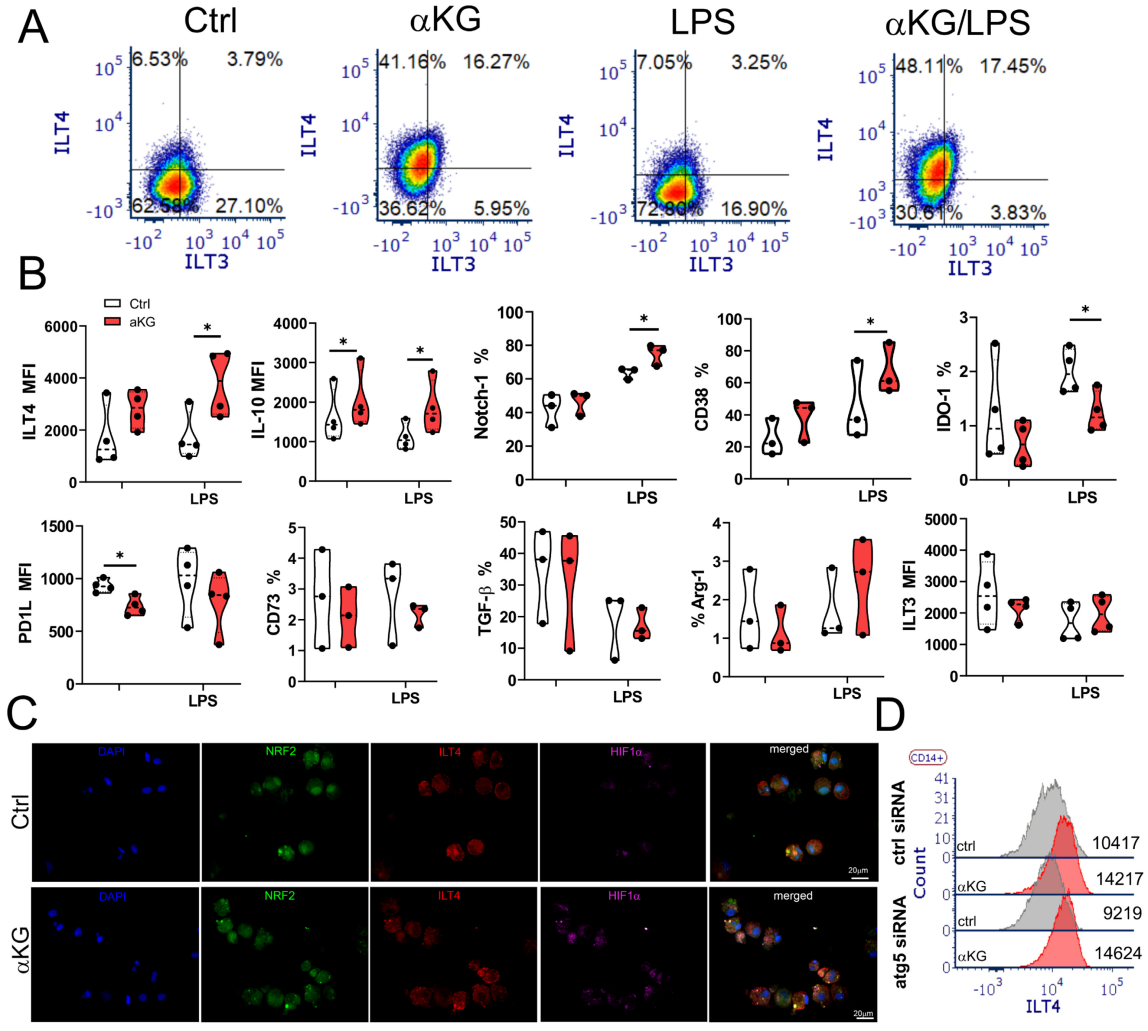
** Suppressive phenotype of moMDSCs induced by αKG.** moMDSCs were differentiated for 5 days with GM-CSF and IL-6, with or without 30 mM αKG from day 0, and with or without LPS for the last 16-18 h. **A)** Representative analysis of ILT3 and ILT4 co-expression and **B)** Summarized data showing % of cells expressing indicated marker or its mean fluorescent intensity (MFI), are shown as violin plots with median and quartiles, with dots of independent experiments. *p < 0.05 as indicated (RM-ANOVA, Dunnett's post-test). **C**) Epifluorescent image of LPS-stimulated control (ctrl) or αKG-treated moMDSCs after staining with NRF-2 Alexa fluor 488, ILT4-PE, HIF-1α Alexa fluor 647 and DAPI. **D)** A representative analysis of ILT4 expression on LPS-stimulated moMDSCs pretreated with Atg5-siRNA or scrambled (ctrl) siRNA for 24h, followed by the treatment with αKG and LPS, as indicated.

The phenotype analysis of αKG-moMDSCs indicated that these cells express higher levels of suppressive markers CD38 and Notch-1, after the LPS stimulation, as compared to corresponding control moMDSCs (Figure 6). Besides increased IL-10, αKG-moMDSCs displayed substantially increased expression of ILT-4 after the LPS stimulation (Figure 6A, B). ILT-4 expression was clearly allocated on moMDSCs displaying both reduced nuclear localization of Nrf-2 and increased HIF-1α expression after the αKG treatment (Figure 6C). However, unlike IL-10, increased expression of ILT-4 in αKG-moMDSCs was not impaired after Atg5 silencing (Figure 6D). αKG-moMDSCs displayed reduced levels of IDO-1 and PD1L compared to control cells, whereas other molecules involved in immunosuppression (CD73, TGF-β, Arg-1 and ILT-3) were not modified significantly (Figure 6B).

**Figure 7:**
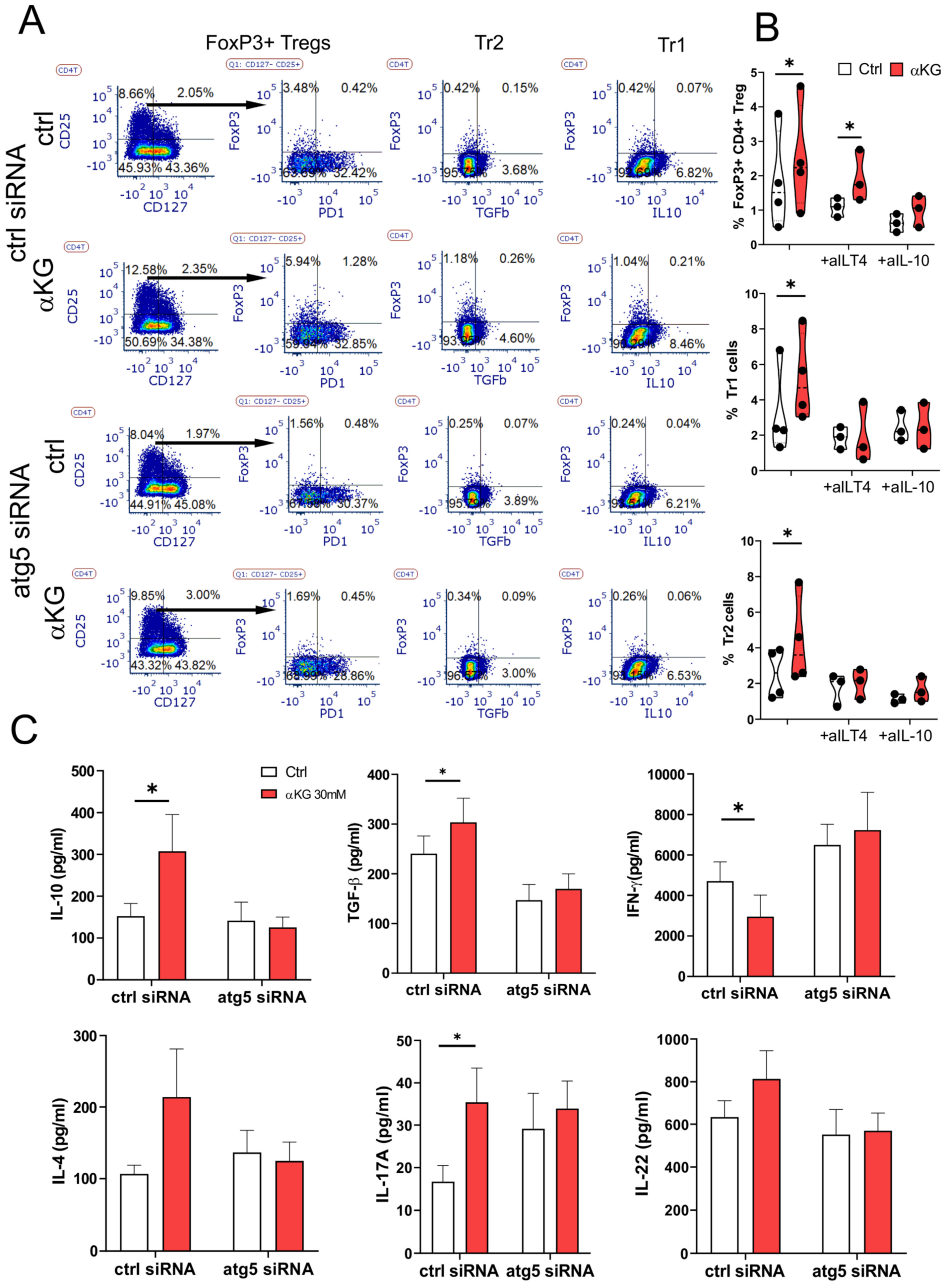
** Regulatory T cells induction by αKG-moMDSCs.** MoMDSCs generated with αKG (30 mM) or without it (ctrl), and stimulated with LPS (100 ng/ml) were co-cultivated with allogeneic T cells (1x10^5^/well) (moMDSCs: T cells 1:4 ratio) in the presence of CD3/CD28 Dynabeads (1:8 beads: T cell ratio) and IL-2 (2 ng/ml) for 6 days. **A)** A representative analysis of conventional Tregs (CD127^-^CD25^+^FoxP3^+^), induced Tr1 (IL-10^+^FoxP3^-^) and Tr2 cells (TGF-β^+^FoxP3^-^) within CD4^+^ T cells after the co-cocultures with moMDSCs pre-treated with Atg5-siRNA or scrambled (ctrl)-siRNA for 24h prior to αKG, as described, from one experiment, out of three with similar results. **B)** Summarized data on % of FoxP3^+^ Tregs, Tr1 and Tr2 cells after the cultures with moMDSCs carried out in the presence of anti-ILT4 Ab, anti-IL-10 (2 µg/ml) or irrelevant Ab, is shown as violin plots with median and quartiles, with dots of independent experiments. **C)** Concentration of cytokines measured in supernatants of cultures performed as in (A) are shown as mean ± SEM (n=3). *p < 0.05 as indicated (RM-ANOVA, Dunnett's post-test).

Based on these data we tested which types of Tregs are induced by αKG-moMDSCs. The analysis of conventional Tregs, according to CD4^+^CD127^-^CD25^+^FoxP3^+^ phenotype (Figure 7A), showed that αKG-moMDSCs increased the proportion of FoxP3^+^ Tregs, majority of which lacked PD1 exhaustion marker. Moreover, this effect αKG-moMDSCs could not be blocked with anti-ILT4 antibody (Figure 7B). However, the induction of conventional Tregs in co-cultures with αKG-moMDSCs was inhibited completely in the presence of blocking anti-IL-10 Ab, or by using αKG-moMDSCs silenced for Atg5 (Figure 7A, B). We also analysed the induced Treg subsets, i.e. CD4^+^FoxP3^-^ Tregs producing IL-10 (Tr1) or TGF-β (Tr2), and also found their increased proportion in co-cultures with αKG-moMDSCs relative to control moMDSCs. The induction of both Tr1 and Tr2 subsets by αKG-moMDSCs could be blocked in the presence of anti-ILT4 Ab, suggesting that ILT4 is involved in their induction. However, their induction was also dependent on Atg5. Namely, when the co-cultures contained αKG-moMDSCs with silenced Atg5, or by conducting the co-cultures in the presence of anti-IL-10, we observed that the percentage of Tr1 and Tr2 cells was similar between the control moMDSCs and αKG-moMDSCs (Figure 7A, B). These results suggested that the induction of autophagy is dispensable for ILT4 expression by αKG moMDSCs, but IL-10 production was still required for the induction of Tr1 and Tr2 cells. Accordingly, we observed increased concentrations of IL-10 and TGF-β in T cell co-cultures with αKG-moMDSCs compared to co-cultures with control moMDSCs, but only when Atg5 was intact (Figure 7C). Interestingly, the silencing of Atg5 also diminished αKG-moMDSCs' capacity to reduce IFN-γ and up-regulate IL-4 in co-cultures with T cells, but also their capacity to up-regulate IL-17 production (Figure 7C).

### αKG-induced autophagy regulates OXGR-1-mediated ROS in moMDSCs and Th17 induction

**Figure 8:**
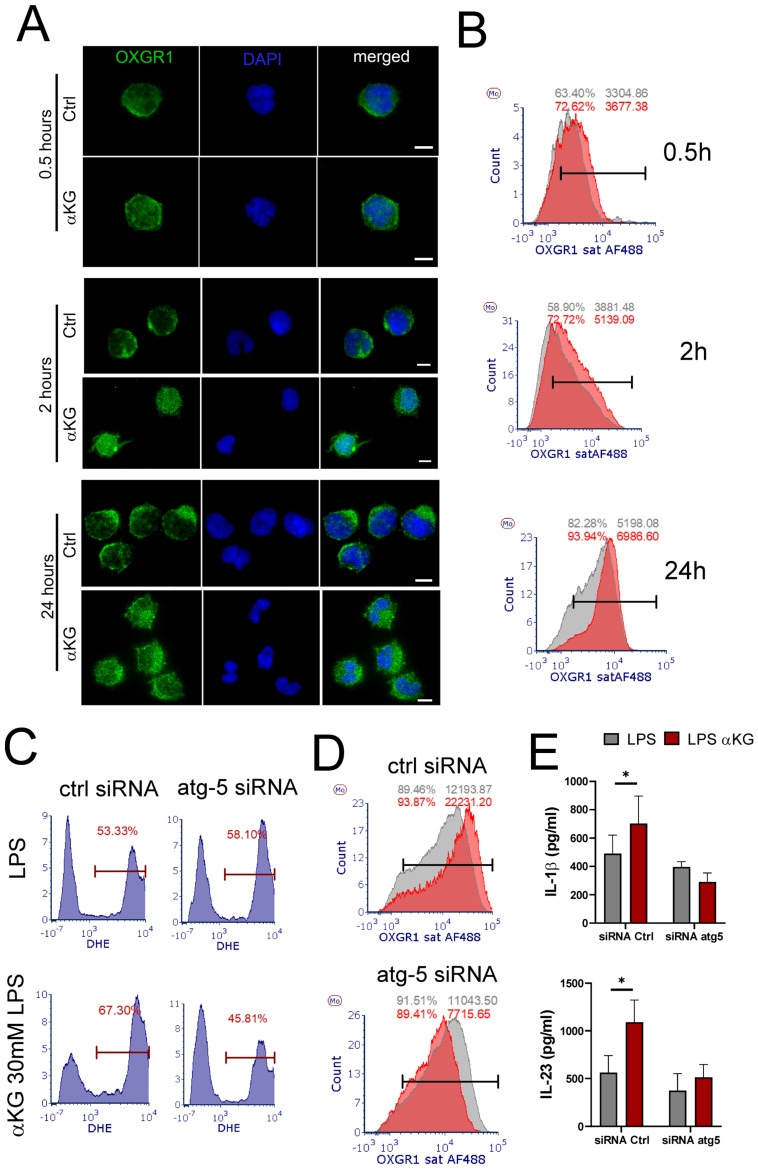
** Role of Atg-5 in OXGR1 expression.** CD14^+^ monocytes were differentiated to moMDSCs with GM-CSF/IL-6 either in the presence of αKG or without it (ctrl) for 4 days and then treated with LPS for the next 16-18 h.** A)** Epifluorescence images of cells collected 0.5h, 2h or 24h after the initial cultures and stained with anti-OXGR1 Ab, and anti-IgG:Alexa 488 and DAPI. **B)** Representative flow cytometry analysis of surface OXGR1 expression in control (grey histograms) and αKG-treated (red histograms) cells collected as in (A). **C)** Representative ROS measurements by DHE staining in moMDSCs pretreated with 1 µg/mL siRNA^atg5^ or control scrambled siRNA (encapsulated in LNPs with 0.5 µg/mL ApoE, Supplement Figure S7) for 24 h, and then differentiated with GM-CSF and IL-6, either with or without 30 mM αKG for 5 days and LPS for the last 16-18h. **D)** Representative analysis of OXGR1 expression in LPS-treated moMDSCs after the cultures as in (C). **A-D)** Representative data is shown from one donor, out of three different donors with similar results. **E)** Concentrations of IL-1β and IL-23 were measured in supernatants of cultures prepared as in (C). Data is shown as mean ± SEM (n=3). *p<0.05 as indicated (RM-ANOVA, Dunnett's post-test).

Atg5-mediated regulation of IL-10 production could explain the observed effects on IFN-γ (Th1) and IL-4 (Th2) polarization (Figure 5F), but it was insufficient to explain how Atg-5 regulates IL-17 polarization in these co-cultures. To assess this, we analysed the expression pattern of OXGR1 during the differentiation of moMDSCs. The epifluorescence microscopy showed that control cells exhibited predominantly membranous localization, characterized by sharp peripheral staining (Figure 8A). In contrast, αKG treatment induced a redistribution of OXGR1, resulting in increased intracellular fluorescence and more widespread expression throughout the cells. Flow cytometry analysis showed that αKG treatments increased the surface expression of OXGR1, as compared to control cells, and that overall expression of OXGR1 (i.e. MFI) increased during the differentiation of moMDSCs (Figure 8B). Interestingly, the analysis of ROS and OXGR1 expression in Atg5-silenced moMDSCs suggested that Atg5 is necessary for both αKG-induced upregulation of OXGR1 expression and ROS potentiation in LPS-treated moMDSCs (Figure 8 C, D). Moreover, αKG-induced induction of IL-1β and IL-23 was completely diminished in Atg-5-silenced cells, as compared to cells pre-treated with scrambled siRNAs (Figure 8E). These results suggested that αKG-induced Atg-5 is critical, not just for Treg inducing properties of moMDSCs, but also for their capacity to induce Th17 cells, mostly via regulation of OXGR1 expression.

## Discussion

αKG was described as having potentially beneficial effects in cancer therapy due to its capacity to modulate chromatin-modifying enzymes 44, reducing cancer's metabolic adaptability to glucose deprivation 45, potentiating radio-sensitization 46, chemotherapy 47, and susceptibility to CKIs targeting PD1L/PD1 axis 25. However, several studies focusing on the effects on immune cells pointed that αKG can induce potentially adverse immunological effects in cancer therapy 26,27,29. However, the effects of αKG on MDSCs, as crucial cells of TME and chronic inflammation driving tumour progression, have not been investigated to date. Here we used a model of GM-CSF/IL-6-induced moMDSCs, which was previously demonstrated as a reliable model for studying moMDSCs biology (9, 48). These cells are susceptible to activation of suppressive mechanisms *in vitro* and *in vivo* 9,49, mirroring their two-step activation paradigm 4. To additionally evaluate this *in vitro* model, we compared the transcriptomic profiles of moMDSCs generated with GM-CSF/IL-6 and stimulated or not with LPS, with the *ex vivo* transcriptomic profiles of MDSCs from studies with COVD-19 50 and diffuse large B-cell lymphoma 51 patients (Supplementary Figure S10). Principal component analysis (PCA) suggested that moMDSCs generated *in vitro* from healthy donors do not differ significantly from *ex vivo* MDSCs profiles obtained from blood cells of healthy donors within these studies.

However, *in vitro* moMDSCs differed significantly from *ex vivo* MDSCs obtained from blood of COVID-19 or DLBC patients. These results suggest that the tissue microenvironment critically shapes MDSCs favouring their pathogenic transcriptomic profile. In line with this, van Wigcheren *et al*. 48 obtained similar distances in PCA between *in vitro* moMDSCs and *ex vivo* MDSCs from head-and-neck squamous cell carcinoma patients, although their phenotype and suppressive functions were mutually similar. By relying on functional similarities between *in vitro* model and *ex vivo* analysis, these authors found that PI3K-Akt signalling is critical for down-regulation of HLA-DR, ROS induction and suppressive properties of MDSCs, which is also in line with our observations on αKG effects. Interestingly, the plasma levels of αKG were recently found to be significantly elevated in patients with long COVID-19 52, which contributes further to our previous finding that moMDSCs are the major drivers of immune dysregulation in this disease 2. However, whether similar metabolite changes and moMDSCs activation occur in tumors or chronically inflamed tissues requires further studies in human patients and relevant animal models. Here we demonstrated for the first time that non-esterified αKG, unlike D-αKG derivate, expands GM-CSF/IL-6-induced differentiation of CD14^+^HLA-DR^low/-^ moMDSCs, but also potentiates their capacity to produce both IL-10 and IL-1β upon activation via TLR-4, thus potentially contributing to chronic inflammation. Thereby, we showed that the pro-inflammatory and immunosuppressive functions of moMDSCs are regulated in a crosstalk between OXGR1-mediated inflammasome priming/licencing and Atg-5-mediated autophagy, respectively.

First, αKG and D-αKG were applied in non-toxic immunomodulatory doses, after testing dose ranges usually applied for *in vitro* studies. Namely, previously the non-esterified αKG were shown to decrease viability of osteoblast MC3T3-E1 cells 34 and cancer HT-29 cells 35 in doses ≥50 mM, whereas in human hFOB1.19 cells the toxic doses were ≥75mM 34, which is in line with the observations in this, and our previous study 29. In contrast, D-αKG displays much higher cytotoxicity in cultures of HSC-T6 and BRL-3A cell lines 53. Madala *et al*. 54 showed that doses ~ 6.6 mM of D-αKG are particularly toxic for tumor cells with high energy demands (>50% toxicity), which is in line with our observation in PBMCs cultures. The *in vitro* doses of αKG and D-αKG used are much higher than those usually observed *in vivo* upon αKG supplementation 25, but they were depicted to better represent the concentrations used in published data and because they provided measurable *in vitro* effects. The anti-proliferative effects of non-esterified αKG in PHA-PBMCs cultures correspond to its capacity to up-regulate anti-inflammatory cytokines (IL-10 and IL-4), and suppress pro-inflammatory cytokines (IFN-γ, TNF-α and IL-5) 29. In contrast, Matias *et al*. 55 showed that D-αKG fuels TCA cycle, OXPHOS and fatty acid generation in T cells, leading to activation of T-*bet* and RORc-mediated transcription of proinflammatory cytokines. The activated T cell phenotype induced by D-αKG treatment in our experiments could be related to these specific metabolic effects. Parker *et al*. 32 previously demonstrated that esterified αKG analogs rapidly hydrolyze in aqueous media and exhibit spurious αKG-independent effects on cellular metabolism, including extracellular acidification and analog-specific inhibitory effects on glycolysis or mitochondrial respiration. Contrary to the previous views, non-esterified αKG is rapidly internalized by various cell types within 24 h 32. We demonstrated that it is stable in cell-free media, and that ~50% is taken up by human monocytes within 48 h 29. These results indicate that esterified αKG predominantly act via mitochondrial TCA 32, whereas non-esterified αKG seems to can act both via surface receptor and intracellularly. However, to avoid any non-specific effects of αKG ester groups in our study, we further focused only on non-esterified αKG.

Myeloid cells express OXGR1 21 which can be activated by αKG (56, 57), and more potently by itaconate 22. Here we showed that in moMDSCs αKG induced a transient phosphorylation of Erk and Akt via OXGR1, followed by their sustained dephosphorylation, increased ROS, IL-1β production and HLA-DR down-regulation. In line with this, Zheng *et al*. 40 showed that the transient phosphorylation of Erk, 10 minutes upon itaconate or αKG treatments, induces β-Arestin-mediated redistribution of OXGR1 receptor expressed ectopically on HEK293 cells, although the downstream trafficking fate of internalized OXGR1 was not studied further. The described OXGR1 redistribution could explain our observation on a wider OXGR1 expression in αKG-moMDSCs as compared to control moMDSCs, but the induction of OXGR1 expression probably involved additional mechanisms.

Previously, Yuan *et al*. 57 demonstrated that exogenous αKG upregulates OXGR1 mRNA and protein expression in adrenal glands cells via NF-kB activation. Our data extends this by demonstrating Atg5 dependence of these phenomena in moMDSCs, representing a novel link. Interestingly, Paddar *et al*. 58 recently pointed to a noncanonical role of Atg-5 in modulation of retromer's core components (VPS26, VPS29, and VPS35), thus regulating GLUT1 endosomal sorting and its surface expression. Considering that similar mechanisms of recycling are involved in different GPCRs 59, it is possible that αKG-increased OXGR1 expression by activating OXGR1 transcription, as well as by increasing its recycling via Atg5, as this could be blocked by Atg5 siRNAs, but this hypothesis requires further investigations. However, a direct link between OXGR1 signaling/ recycling and Erk/Akt dephosphorylation in moMDSCs still remains unknown. Zhang *et al*. 27 showed recently that OXGR1 knockdown in THP-1 and raw264.7 macrophages negated the impact of αKG-induced downregulation of MHC-II related molecules, which aligns with our data. However, these authors observed an increased Erk phosphorylation in macrophages 24h after the treatment with αKG in tumour conditioning medium 27, suggesting that the dynamics of Erk and Akt phosphorylation may be contingent upon cell type, as well as microenvironmental cues. In this context, we used GM-CSF and IL-6 which both activate Erk and Akt in moMDSCs 60,61, and these kinases participate in intricate positive and negative feedback loops regulating their own activation status via activation of phosphatases 62,63. Therefore, it is possible that additional Erk and Akt activation via OXGR1, modulated the activation of these kinases via downstream negative feedback loops, which requires further investigations. According to our findings, αKG treatment was shown to reduce the activation of Erk1/2 in osteoblast cell lines 23,35, as well as the activation of Akt in human monocytes by activating prolyl hydroxylase-2 (PHD2) which hydroxylates pAkt and label it for ubiquitination 64. A reduced activation of Erk1/2 and Akt was previously linked with a reduced nuclear import of Nrf2 41,65. Thereby, Nrf2 downregulation in αKG-moMDSCs have probably lowered the overall anti-oxidative protection in αKG-moMDSCs, thus facilitating the increased ROS levels. Unlike HO-1, SOD-1 expression was increased, probably in Nrf2-independent manner 66,67. Thereby, we showed additionally that the increased ROS in αKG-moMDSCs is most probably related to an altered OXPHOS in mitochondria of αKG-moMDSCs. Accordingly, αKG was shown to modulate IDH2 activity 68, which is critically involved in the regulation of OXPHOS mitochondrial ROS 69. Besides, HIF-1α-mediated inhibition of pyruvate uptake by mitochondria was described as a master switch from OXPHOS to increased glycolysis 42, which also supports our findings on increased HIF-1α, reduced OXPHOS and increased glycolysis in αKG-moMDSCs. In cancer cells, αKG is often a substrate for PHD2 which induce HIF-1α degradation 70. However, αKG can also activate P4HA1, displaying 3-fold higher affinity to αKG than other PHDs, but it inhibits HIF-1α degradation by hydroxylation 71. Interestingly, P4HA1 is highly expressed in non-classical monocytes 21, suggesting that in moMDSCs P4HA1 is a more likely target of αKG, though this requires further investigations.

IL-1β^+^ myeloid cells in tumour were recently described as major drives of cancer progression and inflammation 6. In this context, the role of OXGR1 in IL-1β-mediated inflammation by moMDSCs could be adverse in tumour therapy, and its silencing via LNPs delivering OXGR1-siRNAs a promising therapeutic approach. ROS-mediated potentiation of IL-1β production is a well-recognized phenomenon. Namely, NLRP3 inflammasome is necessary for the conversion of pro-IL-1β to IL-β, and its activation is regulated by a two-step process of priming/licensing and assembly 72. Thereby, mitochondrial ROS was described as critical for both steps of inflammasome activation 73,74. Tannahill *et al*. 75 previously demonstrated that the esterified αKG reduces glycolysis/OXPHOS ratio and HIF-1α-mediated transcription of IL-1β, and its production by LPS-stimulated bone-derived macrophages, indicating their M2 polarization. These findings highlight the differences in the effects of esterified and non-esterified αKG on immune cell metabolism and point to an additional mechanism by which an increased HIF-1α can upregulate LPS-induced IL-1β production in myeloid cells. Besides, dietary supplementation of mice with non-esterified αKG was shown to induce M2 polarization of macrophages, reduced glycolysis/OXPHOS ratio in these cells and lowered IL-1β expression in a model of DSS-induce colitis 76. These findings indicate that the response of macrophages to αKG is different from the response of moMDSCs, which could be expected from their fundamental differences in cell-type-specific metabolic requirements, phenotypes and functions 49,77. A consequence of OXGR1-mediated increase of IL-1β production by αKG-moMDSCs was their increased capacity to induce Th17 cells. The effects of Th17 on tumor progression depend on multiple factors in TME 78, and recent data indicated their pathogenic role in cancer initiation 79. IL-1β was demonstrated to induce alternative splicing of FoxP3 transcription factor, thus favoring Th17 cell differentiation and IL-17 production 80. Moreover, IL-1β is known to stimulate IL-23 production by myeloid cells, thereby sustaining ROR-γt expression in T cells and Th17 response 39. Interestingly, these authors also demonstrated that the inhibition of autophagy with PI3K inhibitors or siRNAs against beclin-1 and Atg7, potentiates these IL-1β-mediated effects upon TLR stimulation, and that the processes are ROS dependent 39. Complementing these findings, we demonstrated further that silencing of OXGR1-mediated, ROS-mediated induction of IL-1β does not impair autophagy in moMDSCs induced by αKG, whereas Atg-5 silencing abrogates OXGR1/ROS-mediated induction of IL1β in these cells. Such an effect of Atg-5 silencing was probably related to its noncanonical role in receptor recycling 58, as discussed previously. Although we cannot exclude completely that the reduced pAkt and increased ROS contributed to increased autophagy in moMDSCs via reduced mTORC1 phosphorylation 81 and activation of Atg4 82, respectively, OXGR1-independent autophagy induction rather points to other mechanisms involved. The potential mechanism most probably included the upregulation of HIF-1α in moMDSCs, as it was shown that HIF-1α increases the transcription and cytoplasmic accumulation of FOXO1 83, which then induces autophagy by interacting with Atg7 84. Additionally, HIF-1α activates Beclin-1 via increased transcription of BNIP3 and Beclin-1 itself 85. A similar phenomenon was observed in our previous study with αKG-treated moDCs, which displayed increased levels of HIF-1α, phosphorylated FOXO1 (cytoplasmic fraction) and increased autophagy upon αKG treatment, all of which led to their increased immunosuppressive properties 29. Furthermore, HIF-1α-mediated reprograming of monocytes and other myeloid cells was demonstrated as critical for induction of their immunosuppressive properties 86, suggesting that similar mechanisms operate in different monocyte-derived cells.

The key finding in this study was that αKG-induced suppressive properties of moMDSCs are predominantly IL-10-mediated and autophagy-dependent. Previous findings showed that pharmacological inhibition or genetic knockdown of autophagy-related genes (e.g., Atg7, Becn1) resulted in significantly reduced IL-10 release and lower IL-10 mRNA levels, indicating that autophagy acts upstream of IL-10 transcription 87 and Atg5-mediated mechanisms were confirmed as critical for STAT3-dependent suppressive MDSCs properties *in vivo* 88. Yu *et al*. 89 found previously that autophagy induction with rapamycin potentiates IL-10 production by MDSCs and their suppressive properties by inhibiting their negative regulators, i.e. Wnt-signaling and TIM3. Moreover, several papers demonstrated that HIF-1α activity requires functional autophagy 90,91, and that it regulates IL-10 expression coordinately with STAT3 86. MDSCs-derived IL-10 is known to be directly involved in the suppression of Th1 cell polarization, induction of Th2 cells 92 and regulatory T cells 93, all of which lead to tumor progression. Accordingly, targeting autophagy in cancer is considered as one of the most promising therapeutic strategies 94, and we showed that LNPs carrying siRNA for Atg-5 could be beneficial in this sense. Besides conventional FoxP3^+^ Tregs, type 1 and 2 FoxP3^-^ induced regulatory T cells have been shown to contribute to immune suppression in tumors and resistance to CKI 95. Thereby, Tr1 can comprise up to 30% of all tumor-infiltrating lymphocytes in colorectal cancer, and these cells display higher suppressive capacity than FoxP3^+^ Tregs 96. The induction of Tr1 cells requires ILT4/HLA-G interactions, but it is completely dependent on IL-10 97. This is in line with our finding that αKG-moMDSCs induction of Tr1 cells can be blocked by either anti-ILT4 Ab, or by anti-IL-10. TGF-β-producing induced Tr2 cells are also dependent on IL-10 98, but this is the first report showing that ILT-4 is also involved in their induction. Although IL-10 is known inducer of ILT-4 97, we showed that the ILT-4 upregulation in αKG-moMDSCs is not dependent on autophagy and IL-10, suggesting the involvement of alternative mechanisms regulating its expression. Besides regulating cellular metabolism, αKG is known to significantly modulate histone methylations and acetylation 99. Thereby, ILT-4 is tightly regulated by histone acetylation at its core promoter in myeloid cells 100. Therefore, although this molecule is increasingly observed as a promising novel candidate for CKI therapy 101, more studies are needed to understand how its expression is regulated by TCA metabolites in different myeloid cells.

In conclusion, the applied doses of non-esterified αKG exerts a dual role in modulating the functions of GM-CSF/IL-6-induced moMDSCs, a model of moMDSCs in TME. By enhancing moMDSCs immunosuppressive functions through autophagy-dependent IL-10 production, αKG promoted T cell suppression and Treg induction, potentially exacerbating tumour immune evasion mechanisms. Intriguingly, αKG also drives the expansion of moMDSCs and their IL-1β production upon TLR4 stimulation, via Atg-5-dependent OXGR1 up-regulation, and ROS signalling, thereby potentiating Th17 cell expansion. These mechanisms of chronic inflammation could significantly accelerate tumour progression, induce immune paralysis and present highly relevant therapeutic targets of immunotherapy, presuming that they operate *in vivo* as well. Our findings underscore the complex interplay between signalling and metabolic effects of TCA intermediaries in determining moMDSCs plastic response, highlighting the need for cautious application of TCAs in immunotherapy of cancer, and other diseases with MDSC-mediated pathologies. Thereby, targeted strategies for modulation of autophagy and OXGR1-mediated inflammation, and particularly Atg5, seem promising for reducing adverse TME effects, but further research is necessary to assess their application in cancer therapy.

## Supplementary Material

Supplementary materials and methods, figures and table.

## Data Availability

All data is contained within the article and supplementary information, whereas raw data is available on request from the corresponding author on reasonable request.

## Abbreviations

2-DG: 2-Deoxyglucose
Akt: Protein kynase B
AMBRA1: autophagy and beclin 1 regulator 1
AMPK: AMP- activated protein kinase
APC: Allophycocyanin
Arg: arginase
Atg: autophagy related
BECN1: beclin-1
CKIs: checkpoint inhibitors
Cy: Cyanine
DAPI: 4',6-diamidino-2-phenylindole
DCs: dendritic cells
CD: cluster of differentiation
DHE: dihydroethidium
D-αKG: dimethyl- αKG
ECAR: extracellular acidification rate
ECH: Enoyl-CoA Hydratase
ELISA: enzyme-linked immunosorbent assay
Erk: Extracellular signal-Regulated Kinase
FCCP: carbonyl cyanide 4-(trifluoromethoxy) phenylhydrazone
FCS: foetal calf serum
FITC: fluorescein isothiocyanate
GAPDH: glyceraldehyde-3-phosphate dehydrogenase
GM-CSF: granulocyte macrophages colony-stimulating factor
HCl: hydrochloric acid
HIF-1α: hypoxia-inducible factor 1α
HLA: human leukocyte antigen
HMOX1: heme oxygenase 1
HO-1: Hem oxygenase 1
IDO: indolamine-dyoxygenase
IL: interleukin
ILT: imunoglobulin-like transcript
Keap-1: Kelch-like ECH-associated protein
LC3II: membrane converted variant of LC3
LNPs: lipid nanoparticles
LPS: lipopolysaccharide
MACS: magnetic-activated cell sorting
MAP1LC3B: microtubule associated protein 1 light chain 3 beta
MDSCs: myeloid-derived suppressor cells
mo: monocytes
mTOR: mammalian target of rapamycin
MTT: 3-(4,5-dimethylthiazol-2-yl)-2,5-dyphenyltetrazolium
NLRP3: NOD-like receptor family pyrin domain containing 3
NOD: Non-obese diabetic
Notch-1: Neurogenic locus notch homolog protein 1
Nrf: Nuclear Factor Erythroid 2-related Factor
NTA: nanoparticle tracking analysis
OCR: oxygen consumption rate
OXGR1: oxoglutarate receptor
OXPHOS: oxidative phosphorylation
P4HA1: Prolyl 4-hydroxylase subunit alpha-1
PBMC: peripheral blood mononuclear cells
PDH: Prolyl hydroxylase domain
PD-L1: program death ligand 1
PE: phycoerythrin
PerCP: Peridinin-Chlorophyll-Protein
PFA: Paraformaldehyde
PHA: phytohemagglutinin
PI3K: phospho-inositol-3 kinase
PMA: Phorbol 12-myristate 13-acetate
PMN: polymorfonuclear
ROS: Reactive oxygen species
SDS: sodium dodecyl sulphate
siRNA: small interfering ribonucleic acid
SOD1: superoxide dismutase 1
SQSTM1: sequestome-1/p62
TAM: tumour associated macrophages
TCA: tricarboxylic acid
TGF: transforming growth factor
Th: T helper cells
TME: tumour microenvironment
TNF: tumour necrosis factor
Tr1: type 1 regulatory T cells
Tregs: regulatory T cells
αKG: alpha-ketoglutarate (2- oxoglutarate)
