# Supplementary material for: Alpha-ketoglutarate Potentiates IL-1β Production and Suppressive Mechanisms of Myeloid-Derived Suppressor Cells by Altering Redox Metabolism and Inducing Autophagy
Source: Int J Biol Sci. 2026 May 11;22(10):5119–41. doi: 10.7150/ijbs.127309 (PMC13215254; doi:10.7150/ijbs.127309)
Supplement: Supplementary file 1 — Supplementary materials and methods, figures and table. [file ijbsv22p5119s1.pdf]

Supplementary information

Alpha-ketoglutarate potentiates IL-1β production and suppressive mechanisms of myeloid-derived suppressor cells by altering redox metabolism and inducing autophagy

Marijana Milanović[1], Luka Pavlović[2], Marina Bekić[2], Jelena Đokić[3], Marija Stojadinović[4], Dušan Radojević[3], Miodrag Čolić[2, 5, 6], Sergej Tomić[2*]

[1] University of Defence- Medical Faculty of the Military Medical Academy, Belgrade, Serbia

[2] University of Belgrade- Institute for the Application of Nuclear Energy, Department for Immunology and Immunoparasitology, Belgrade, Serbia.

[3] University of Belgrade- Institute for Molecular Genetics and Genetic Engineering, Belgrade, Serbia

[4] University of Belgrade – Faculty of Chemistry, Centre of Excellence for Molecular Food Sciences, Department of Biochemistry, Belgrade, Serbia

[5] Serbian Academy of Sciences and Arts, Belgrade, Serbia

[6] University of East Sarajevo-Medical Faculty Foča, Foča, Bosna and Hercegovina

*Correspondence: Sergej Tomić, Principal Research Fellow, University of Belgrade- Institute for the Application of Nuclear Energy. Address: Banatska 31b, Zemun, Belgrade, 11080, Serbia, Tel: +381 11 2610 126; Fax: +381 11 2618 724; email: sergej.tomic@inep.co.rs

Supplementary Methods

Zymography

MoMDSCs were harvested at 48h or 5 days after the treatment with α-KG. The cells were lysed in RIPA buffer (50 mM Tris-HCl pH 7.4, 150 mM NaCl, 0.25% sodium deoxycholate, 1 mM EDTA, 1% Triton X-100, 0.1% SDS) supplemented with protease (cOmplete™, Roche, Basel, Switzerland) and phosphatase inhibitors (PhosSTOP™, Roche), for 25 min on ice, centrifuged at 13,000×g for 20 min, and the protein concentration in lysate was determined using the Pierce™ BCA Protein Assay Kit (Thermo Fisher Scientific, Waltham, MA, USA). Cathepsin activity was determined in moMDSC cell lysates pooled from two donors in an equal ratio, as described previously [1]. After cell lysis, the samples were mixed with non-reducing denaturing SDS buffer and 16 μg of total protein was loaded into each well of the gel. Protein separation was carried out on a 12 % acrylamide gel containing 1 mg/ml gelatine by electrophoresis. The gel was initially incubated 3 x 10 min in the renaturation buffer (65 mM Tris, 20 % glycerol (V/V) pH 7.4), and then overnight in the activation buffer (0.1 M sodium phosphate buffer, pH 6.0, 0.001 M EDTA, 0.002 M DTT) at 37 ºC. The gel was stained with the Coomassie Brilliant Blue solution (0.2 g of dye, 100 ml of methanol, 20 ml of acetic acid and distilled water to a final volume of 200 ml), for 30 minutes, or until the appearance of white bands on the blue background, which was followed by destaining in 7 % acetic acid.

Flow cytometry

The following antibodies (clones) were used**:** IgG1 negative control-fluorescein isothiocyanate (FITC) (MCA928F), IgG1 negative control-phycoerythrin (PE) (MCA928PE) (all from Bio-Rad Laboratories, Hercules, California, United States); CD14-fluorescein isothiocyanate (FITC) (TUK4); CD1a- Peridinin-Chlorophyll-Protein (PerCP)/Cyanine (Cy) 5.5 (HI149), anti-HLA-DR-Allophycocyanin (APC)/Cy7 (L234), anti-CD206-APC (15-2), anti-CD86-PerCP Cy5.5 (IT2.2), anti-CD205-biotin (NLDC-145), PD-L1-PE (29E.2A3), CCR7-PE (G043H7), CXCR3-FITC (CXCR3-173), CCR6-PerCy5.5 (G034E3), CCR4-APC-Fire750 (L291H4), CCR2-PerCPCy5.5 (K036C2), CD45RA-APC (HI100), TNF-α-APC-Cy7 (Mab11), anti-IL-4-PerCP-Cy5.5 (MP4-25D2), anti-IFN-γ-FITC (4S.B3), IL-17A-PE (BL168), CD8-PE-Cy7 (SK1), CD4-Alexa Fluor 700 (OKT4), anti-CD127-PE (A019D5), CD25-PerCP-Cy5.5 (M-A251), TGF-β-PE-Cy7 (TW4-6H10), anti-IL-10-APC (JES5-16E3), IgG1 negative control-PerCP-Cy5.5 (HTK888), PD1-APC-Cy7 (EH12.2H7), ILT3-PE-Cy7 (ZM4.1), anti-ILT4-APC (42D1), CD73-FITC (AD2), anti-Notch 1-PECy7 (MHN1-519), anti-IL-10-PE (JES5-16E3), anti-CD38-APC-Cy7 (HIT2), anti-Arginase I (Arg1)-PeCy7 (14D2C43), anti-CD69-APC (FN50), anti-HLA-DR-Alexa Fluor 488 (LN3), streptavidin-PerCP-Cy5.5, streptavidin APC, streptavidin PE-Cy7 (all from Biolegend); IgG1 negative control APC (MA5-18093), IL-17A-APC (eBio17B7), HIF-1α:biotin (H1alpha67) (all from Thermo Fisher Scientific), CD40-APC (5C3), anti-FoxP3-Alexa Fluor 488 (236A/E7), Fixable viability stain (FVS) 620, (all from BD Biosciences, San Diego, California, United States); anti-IL-1β-PE (rea1172), anti-IDO-1-APC (700838), anti-TGF-β-PE (9016), anti-LOX-1/OLR1 Alexa Fluor 700 (all from R&D Systems, Minnesota, United States). After incubation of cells with OXGR1 with recombinant rabbit polyclonal anti-OXGR1 antibody (bsm-62646r, Bioss Inc.), the signal was visualized by using secondary anti-rabbit IgG:Alexa 488 (Thermo Fisher Scientific).

Surface staining was performed in PBS/0.01% Na-azide for 45 min at 4°C. The cells were washed in PBS and stained with FVS620 for 15 minutes, and with secondary IgG if needed,

followed by fixation in 0.2% PFA, and in case of intracellular staining, with the saponin-based permeabilization buffer (Biolegend). Antibodies for intracellular cytokines and backbone lineage markers were then used at 1:1000 dilution in the permeabilization buffer for 16h, at 4°C to increase staining specificity and staining yield. For each analysis, dead cells and doublets were excluded according to fixable viability dye (FVS620) and forward scatter (FSC)-A/FSC-H, respectively. At least 10000 cells were gated according to their specific FSC-A/side-scatter (SSC)-A properties. Signal overlap between the fluorescent channels was compensated before each experiment by using compensation beads (ThermoFisher), and the non-specific fluorescence was determined by using appropriate isotype control antibodies. The samples were acquired on an LSR II flow cytometer with 8 colour channels (BD Biosciences) on the day of staining and analysed offline in FCS Express 7 software (De Novo software, California, United States).

Calcium measurements

Intracellular Ca^{2+} levels were determined in non-stimulated and LPS-stimulated moMDSCs, after collecting the cells and loading them with Fluo-4-AM (1 μM, Invitrogen), according to manufacturer's protocol. The negative control sample was washed in Ca^{2+}-free PBS and treated with EDTA (1 mM) for 10 minutes, before Fluo-4 loading and analysis on Flow cytometer (BD LSR II).

Total RNA extraction and mRNA sequencing

Total RNA was extracted from MDSCs, generated from 3 donors and were or not stimulated with LPS, using the GeneMATRIX Universal RNA Purification Kit (EURx, Gdańsk, Poland), according to the manufacturer's instructions. RNA quantification was performed using a Qubit fluorometer (Invitrogen, USA). Library preparation and mRNA sequencing were performed in paired-end mode (PE150) on the Illumina NovaSeq 6000 at Novogene (Cambridge, United Kingdom). On average, ~32.3 million PE reads (read pairs) per sample were generated.

Comparative transcriptomic analysis

For comparative transcriptomic meta-analysis, publicly available datasets were identified in the Gene Expression Omnibus (GEO) database using the search terms "monocytic", "MDSC", and "Homo sapiens". Only datasets containing high-throughput gene expression profiles of human monocytic MDSCs from patients and healthy controls were included for downstream analysis (Supplement Table 1).

Supplement Table 1. Transcriptomic cohorts included in the meta-analysis.

Cohort	Data source	Sequencing mode	Accession number	PMID	Study
In vitro MDSC	This study	Paired-end	PRJNA1429269	-	This study
Ex vivo MDSC COVID19	GEO	Paired-end	GSE199286	35812392	Manuscript reference 50
Ex vivo MDSC DLBC	GEO	Single-end	GSE287333	39944818	Manuscript reference 51

All datasets were processed independently using the same analysis pipeline on the Data Centre in Kragujevac (Serbia) via secure remote access. For consistency across cohorts, only forward reads were retained from PE libraries, as some datasets were generated using single-end (SE) sequencing. Briefly, raw sequencing reads were quality-checked using FastQC, and adapter sequences were removed and reads trimmed using Trimmomatic (v0.39). Trimmed reads were aligned to the human reference genome (hg38) using STAR (v2.7.11b). The resulting alignment files were converted to BAM format and sorted by genomic coordinates using SAMtools (v1.20) prior to read quantification with featureCounts. All downstream analyses were performed in R using the following packages: DESeq2 (design = ~ Study, to account for batch effects), ggplot2, vegan, apeglm, org.Hs.eg.db, AnnotationDbi, msigdbr, fgsea and readr. Genes with fewer than 10 reads in at least three samples were removed from the dataset. Variance-stabilized expression data obtained through the DESeq2 pipeline were used to calculate sample-to-sample distance matrices (using Euclidean distance by default) and to perform Principal Component Analysis (PCA). Differences in global transcriptomic profiles between groups were assessed using PERMANOVA implemented in the vegan R package (function adonis2). The analysis was performed with 999 permutations. Pairwise PERMANOVA comparisons between groups were conducted to evaluate differences between individual group pairs. Resulting p-values were corrected for multiple testing using the Benjamini-Hochberg false discovery rate (FDR) method.

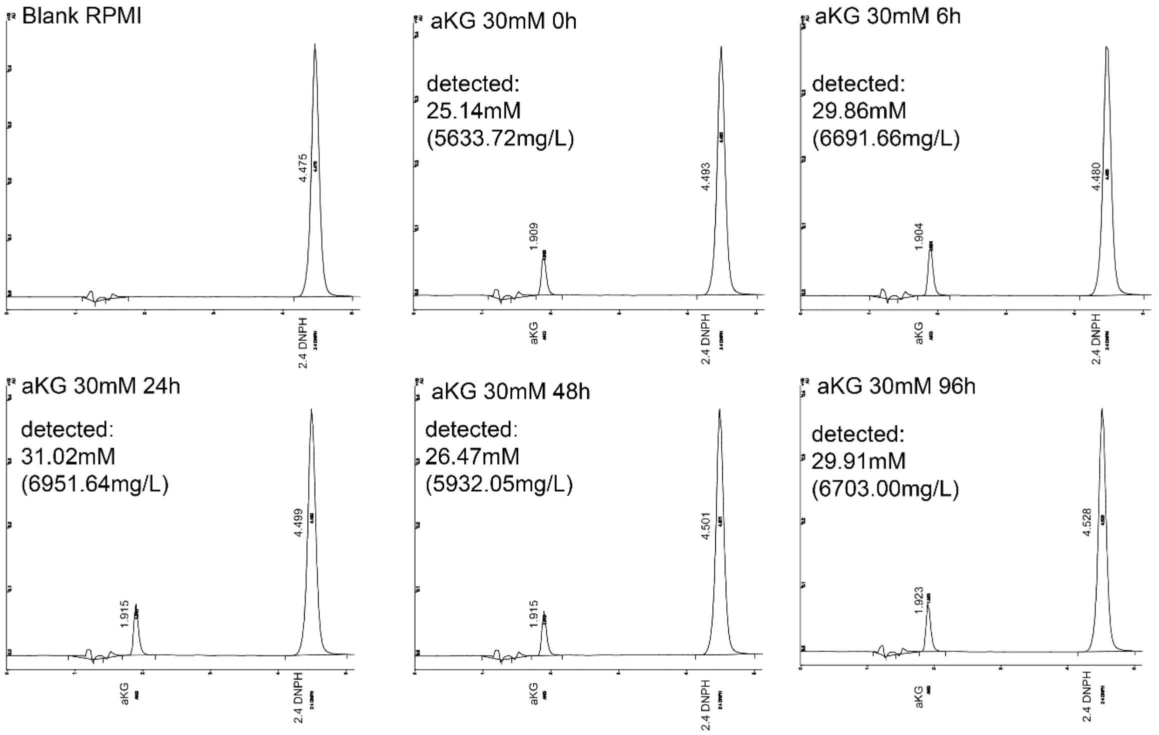

Supplementary Figure S1. α-KG was dissolved in cell-free RPMI medium at 30mM, and the solution was incubated at 37ºC for 0h - 96h, as indicated. The concentrations of α-KG were determined using HPLC, after derivatization with 2,4-dinitrophenylhydrazine (2,4-DNPH), according to the method described previously [2]. The separation was performed on a Zorbax XDB -C8 (4.6 X 150 mm, 5 µm) column and the detection was performed at 380 nm.

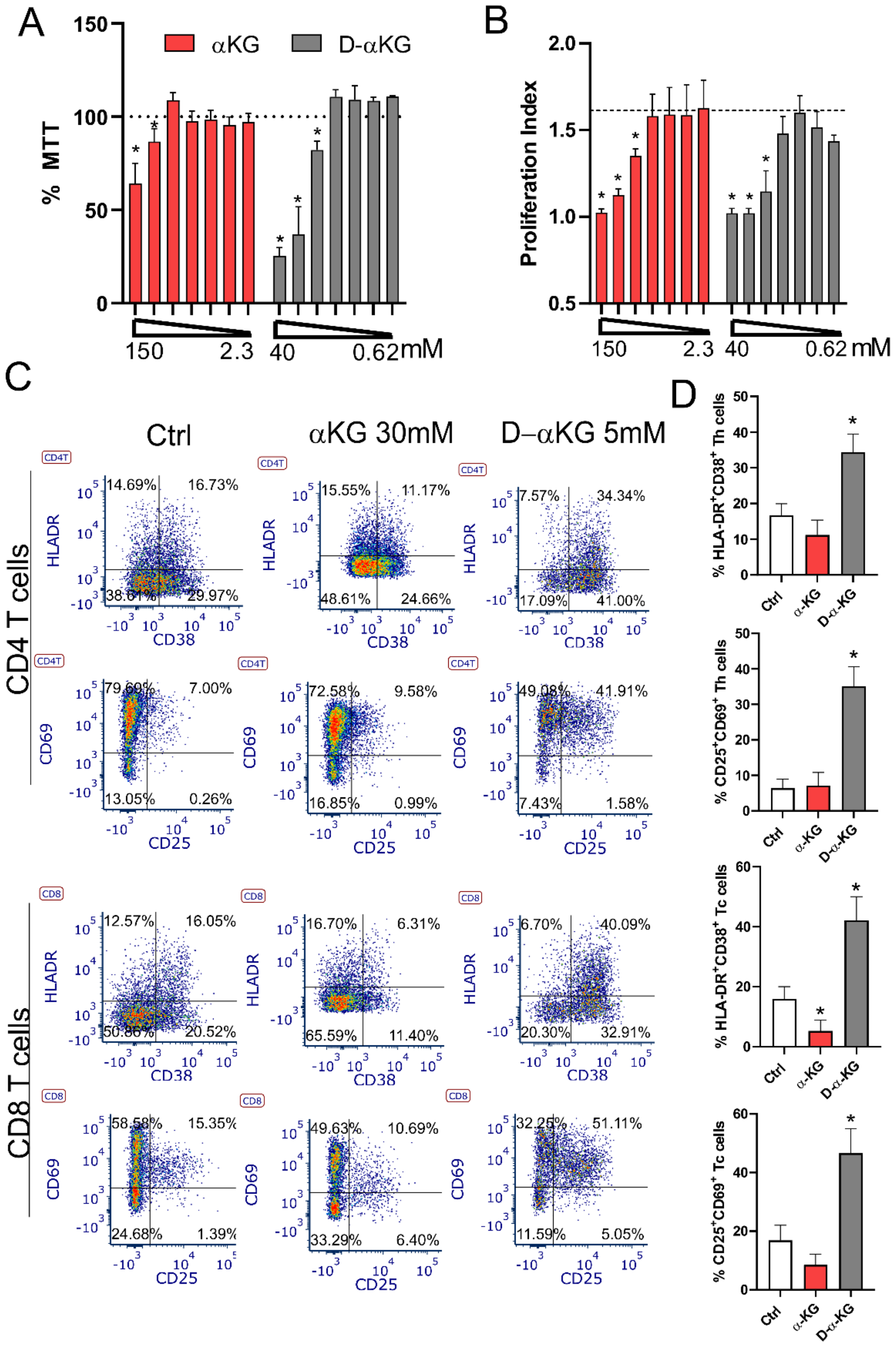

Supplementary Figure S2. Toxicity and immunomodulatory effects of α-KG and D-α-KG in PBMC cultures. PBMCs ($3x10^5$/well of 96-wells plate) were cultivated with different doses of α-KG (2.3-150 mM) or D-α-KG (0.62-40 mM) for 72 h. **A)** Relative metabolic activity (MTT%) was determined after the cultures, as described in methods. **B)** PBMCs were labeled with CTFR and stimulated with PHA, following the cultivation with α-KG and D-α-KG and analysis of CTFR dilution by flow cytometry. **C)** Representative analysis of HLA-DR/CD38 and CD25/CD69 expression within CD4+ T cells or CD8+ T cells from PHA-PBMCs cultivated with α-KG (30mM)

and D-α-KG (5mM), and **D)** Summarized data on % of double-positive cells within CD4 (Th) or CD8 (Tc) population. (A, D, C) Data from three independent experiments is shown as mean ± SEM. *$p < 0.05$ as indicated (RM-ANOVA, Dunnett's post-test).

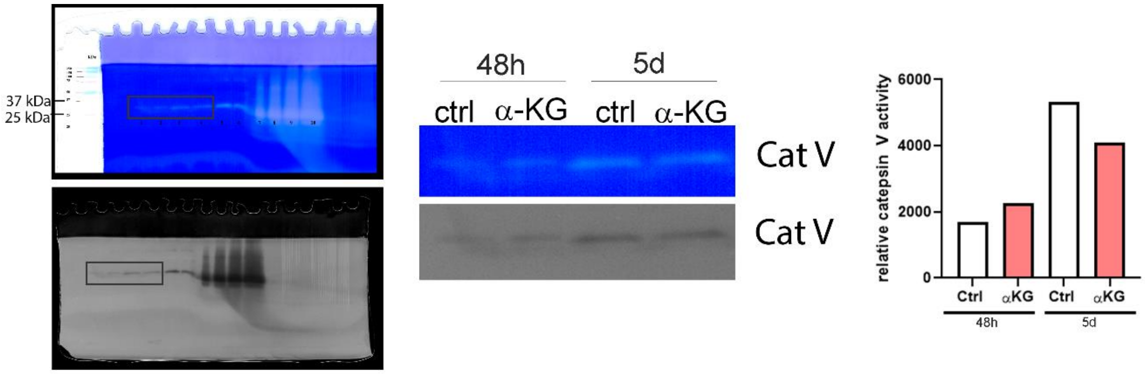

Supplementary Figure S3. Cathepsin activity in moMDSCs treated with α-KG. MoMDSCs were differentiated with GM-CSF/IL-6 in the presence of α-KG (30mM) or its absence (ctrl) for 48 h or 5 days, followed by zymography. Uncut gels are shown (left), enlarged blots (middle) and summarized data obtained upon pooling equimolar concentrations of total proteins from two donors.

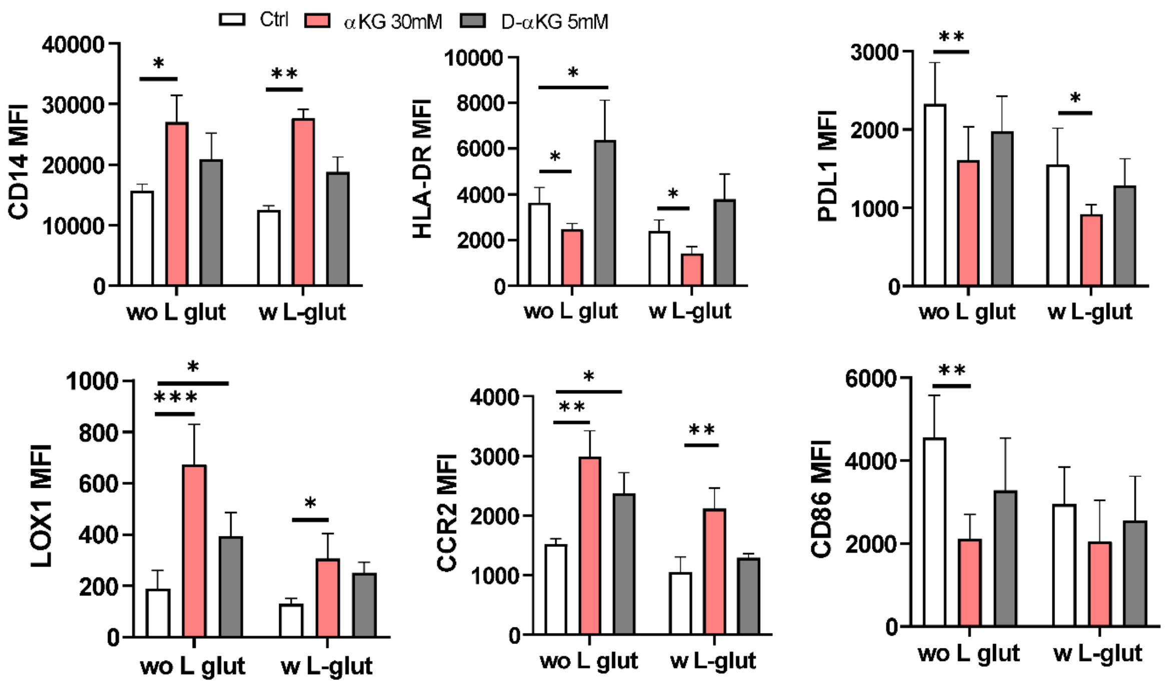

Supplementary Figure S4. Phenotype of moMDSCs differentiated with α-KG and D-α-KG. $CD14^{+}$ monocytes were differentiated into moMDSCs for 5 days with GM-CSF and IL-6, with or without 30 mM α-KG from day 0, 5mM D-α-KG, or their absence (ctrl). The cells were either generated in complete media with (w) L-glutamine (2 mM) or without (wo) it. Mean fluorescence intensity (MFI) for indicated markers, obtained by flow cytometry, is shown as mean ± SEM from three independent experiments. *$p < 0.05$, **$p<0.01$, ***$p<0.005$ as indicated (RM-ANOVA, Dunnett's post-test).

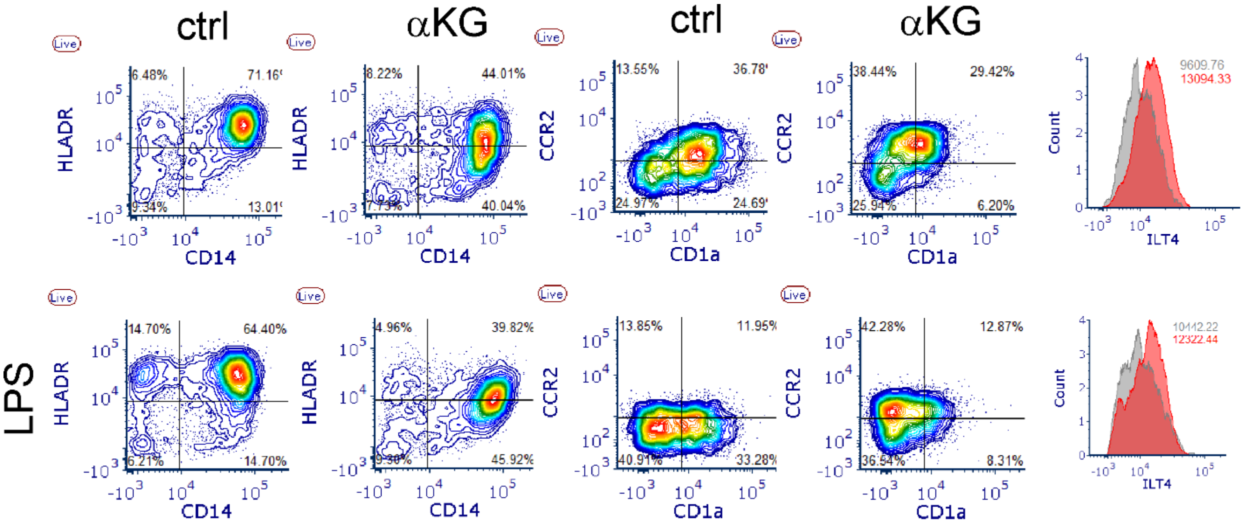

Supplementary Figure S5. Phenotype of moMDSCs differentiated with α-KG for 7 days. CD14+ monocytes were differentiated into moMDSCs for 7 days with GM-CSF and IL-6 in the presence of α-KG (30mM) or its absence and then stimulated with LPS for the next 16 h (lower row) or not (upper row), prior to analysis of CD14, HLA-DR, CD1a, CCR2 and ILT4. Representative contour plots and histograms (grey- ctrl, red- α-KG) are shown from the flow cytometry analysis of cells from one donor, out of two different donors.

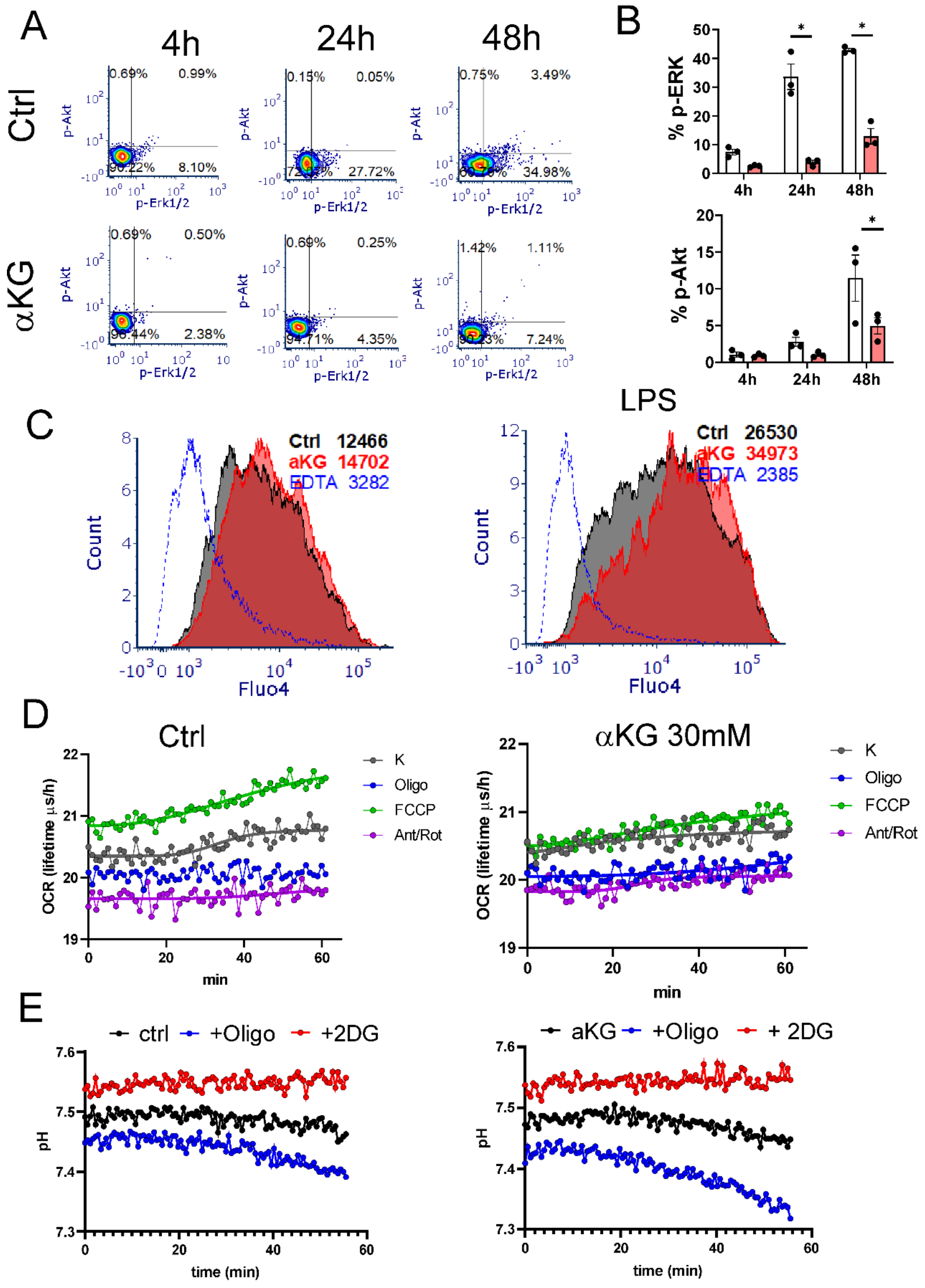

Supplementary Figure S6. Signaling induced by α-KG in moMDSCs. A) Representative analysis of pErk and aAkt in CD14^{+} monocytes treated with GM-CSF, IL-6, and 30 mM α-KG or without it (ctrl) for 4h, 24h or 48h, and **B)** the summarized data from 3 independent experiments are shown. **C)** Representative data on intracellular Ca^{2+} levels in moMDSC differentiated with or without α-KG, and then stimulated or not with LPS for 16-18h, prior to staining with Fluo-4 (and 1mM EDTA for negative control) and flow cytometry analysis. **D)** Representative OCR measurements in moMDSCs differentiated with or without α-KG for 5 days, and threated with Oligomycin (2 µM), FCCP (0.5 µM) or Antimycin / Rotenone (1 µM), and monitoring for 60 minutes (summarized data is shown in Figure 3H). **E)** Representative measurements of ECAR,

here expressed as pH, in moMDSCs as in (D) and treated with Oligomycin (2 μM) or 2-DG (20mM).

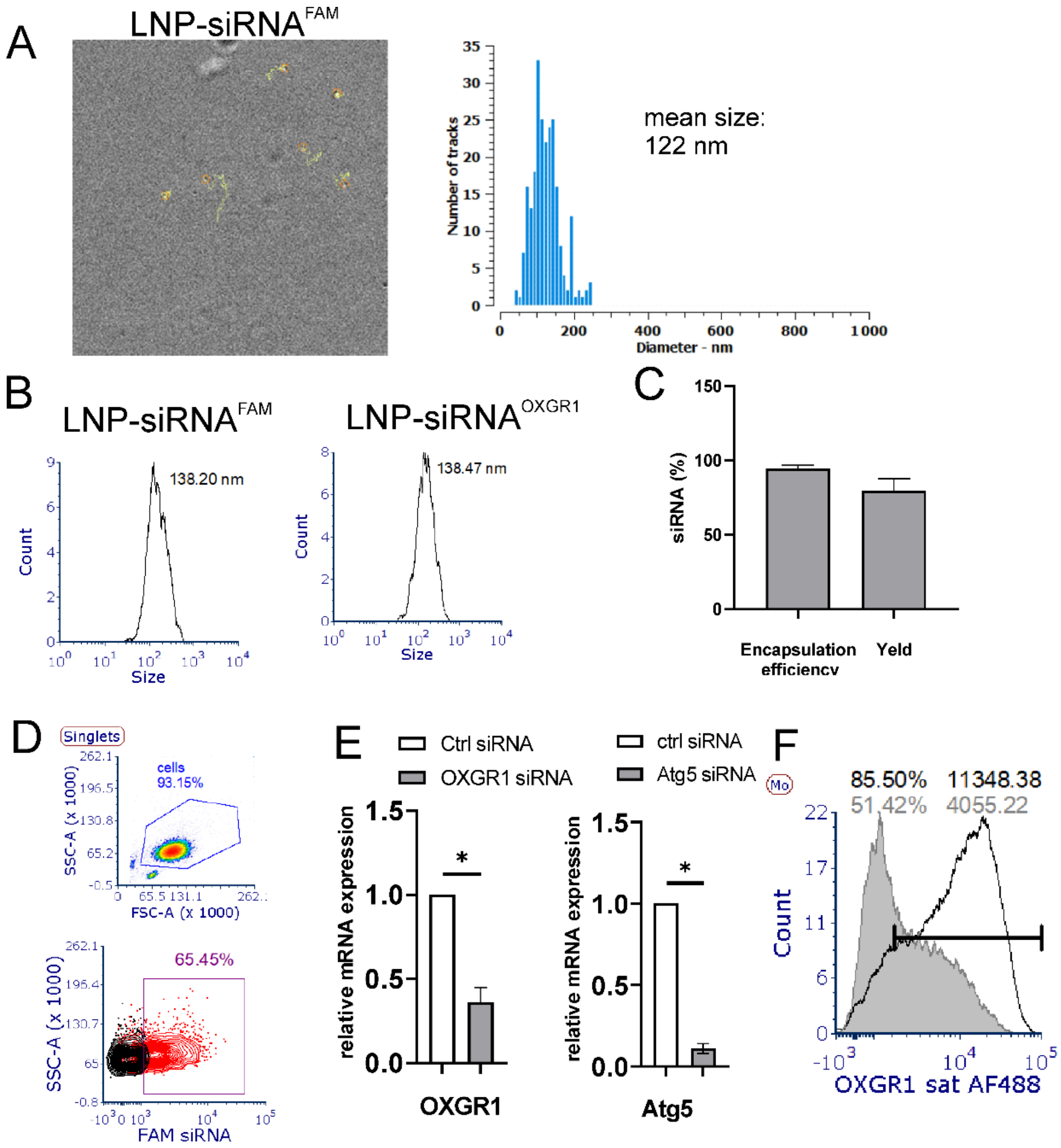

Supplementary Figure S7. Characterization of LNP-siRNAs delivery. siRNA^{FAM}, siRNA^{OXGR1} and siRNA^{Atg5} were encapsulated into LNPs using NanoAssemblr™ Spark™, as described in Methods. **A)** An analysis of LNP-siRNA^{FAM} with Videodrop system with an image (left) containing marked tracks of LNPs used by the system for determination of size distribution (right). **B)** Analysis of LNP-siRNA^{FAM} and LNP-siRNA^{OXGR1} by NTA (ZetaView) with indicated mean LNP size. **C)** Encapsulation efficacy and siRNA yield were assessed in RiboGreen assay by using standard concentrations of RNAs and comparing the non-treated (extravesicular siRNA) and Triton-treated (total siRNA) LNP samples. Data are shown as mean % ± SD of 4 different preparations of siRNAs used in experiments. **D)** Internalization of LNP-siRNA^{FAM} was analyzed by flow cytometry after treating the monocytes with 1μg/ml of LNP-encapsulated siRNA^{FAM} (red) and non-fluorescent LNP-siRNA^{OXGR1} (black) for 24h. **E)** Relative OXGR1 and Atg5 mRNA expression in monocytes, treated with 1 μg/ml of LNP-encapsulated scrambled (ctrl) siRNA^{FAM}

(white), siRNA^{OXGR1} or siRNA^{Atg5} as indicated, and then cultivated with GM-CSF/IL-6 for 3 days. **F)** Flow cytometry assessment of surface OXGR1 expression after the treatment with siRNA^{OXGR1} (grey histogram) or scrambled (ctlr) siRNA^{FAM} (white histogram) as in (E), followed by cultivation of cells in GM-CSF/IL-6 for 5 days.

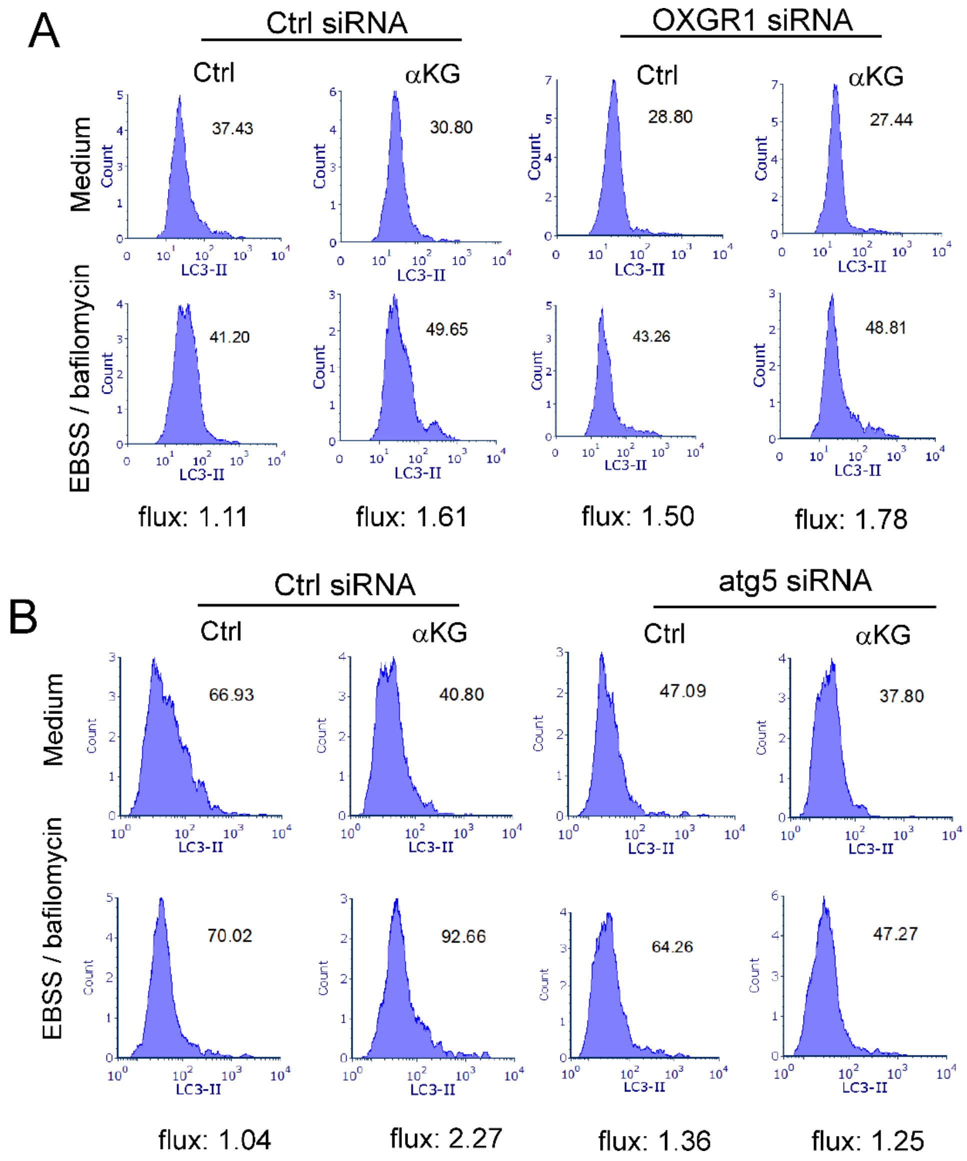

Supplementary Figure S8. Role of OXGR1 and Atg5 in α-KG-induced autophagy. A representative analysis of autophagy flux in moMDSCs pretreated with 1 µg/mL of LNP-encapsulated siRNA^{oxgr1} siRNA^{Atg5}, or scrambled (ctrl) siRNA for 24 h, and then differentiated with GM-CSF and IL-6 with or without 30 mM α-KG for 4 days. LC3 flux was analysed after 4-hour cultivation of cells with bafilomycin in PBS, or in complete medium, and afterwards staining them for membrane-bond LC3 expression and analysing on Cell Muse Analyzer (see Figure 4D for summarized data on siRNA^{oxgr1} experiments).

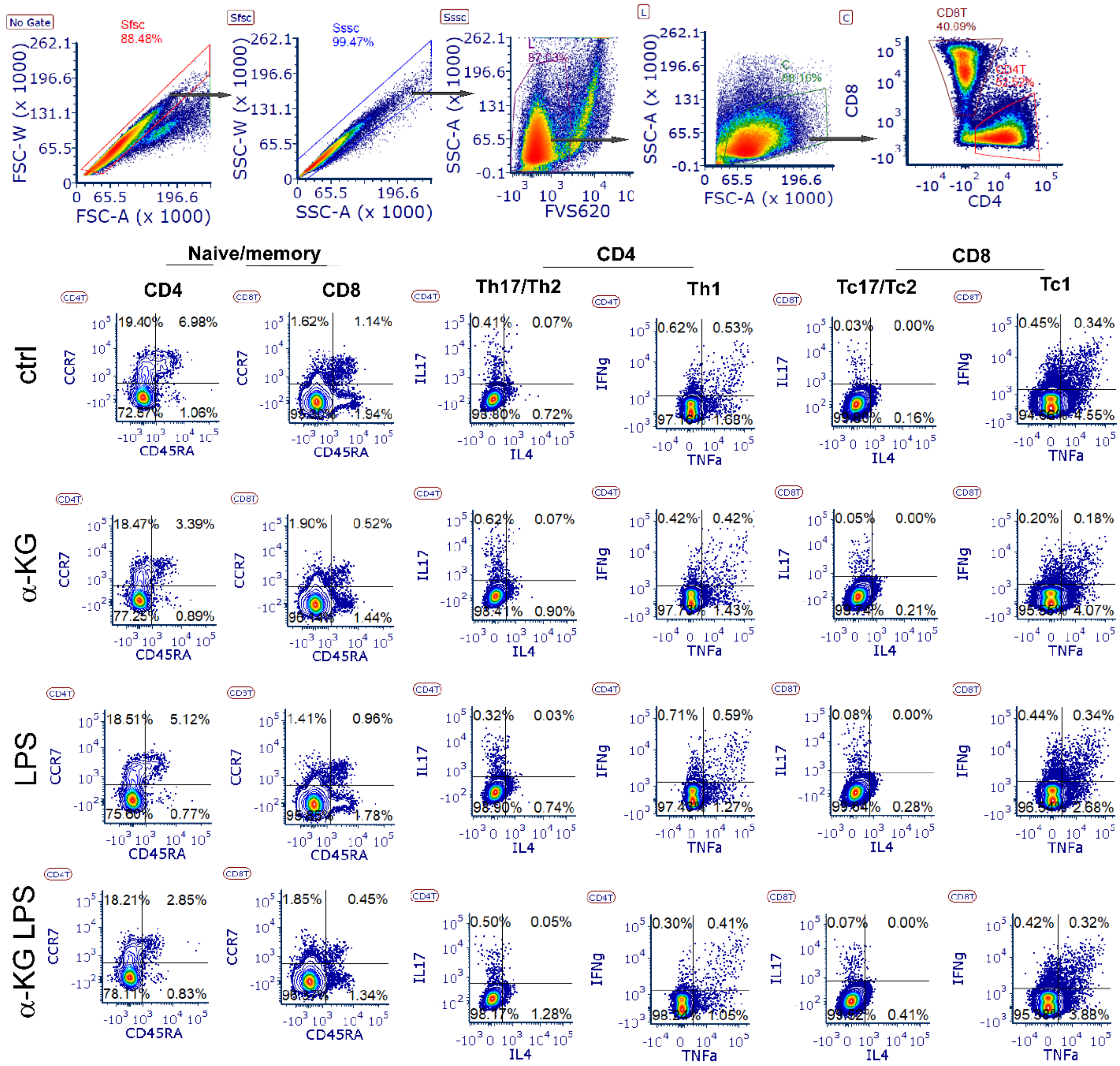

Supplementary Figure S9. Effects of αKG-moMDSCs on T cell polarization. MoMDSCs generated with α-KG (30 mM) or without it (ctrl) and stimulated or not with LPS (100 ng/ml) were co-cultivated with allogeneic T cells (1x10^5/well) in the presence of CD3/CD28 Dynabeads and IL-2 for 5 days, of which the last 4h with PMA/CaI/monensin. A representative gating strategy for CD4 and CD8 T cells is shown, along with the analysis of naïve ($CCR7^+CD45RA^+$), central memory ($CCR7^+CD45RA^-$), effector memory ($CCR7^-CD45RA^-$) or TEMRA ($CCR7^-CD45RA^+$) phenotype. Within CD4+ cells, Th17 cells were identified as IL-17^+, Th2 as IL-4^+ and Th1 cells as IFN-γ^+(TNF-α^+ and TNF-α^-). Likewise, cytotoxic T (Tc)17, Tc2 and Tc1 were determined within CD8 T cells. The summarized data on intracellular cytokines within Th cells is shown in Figure 5F.

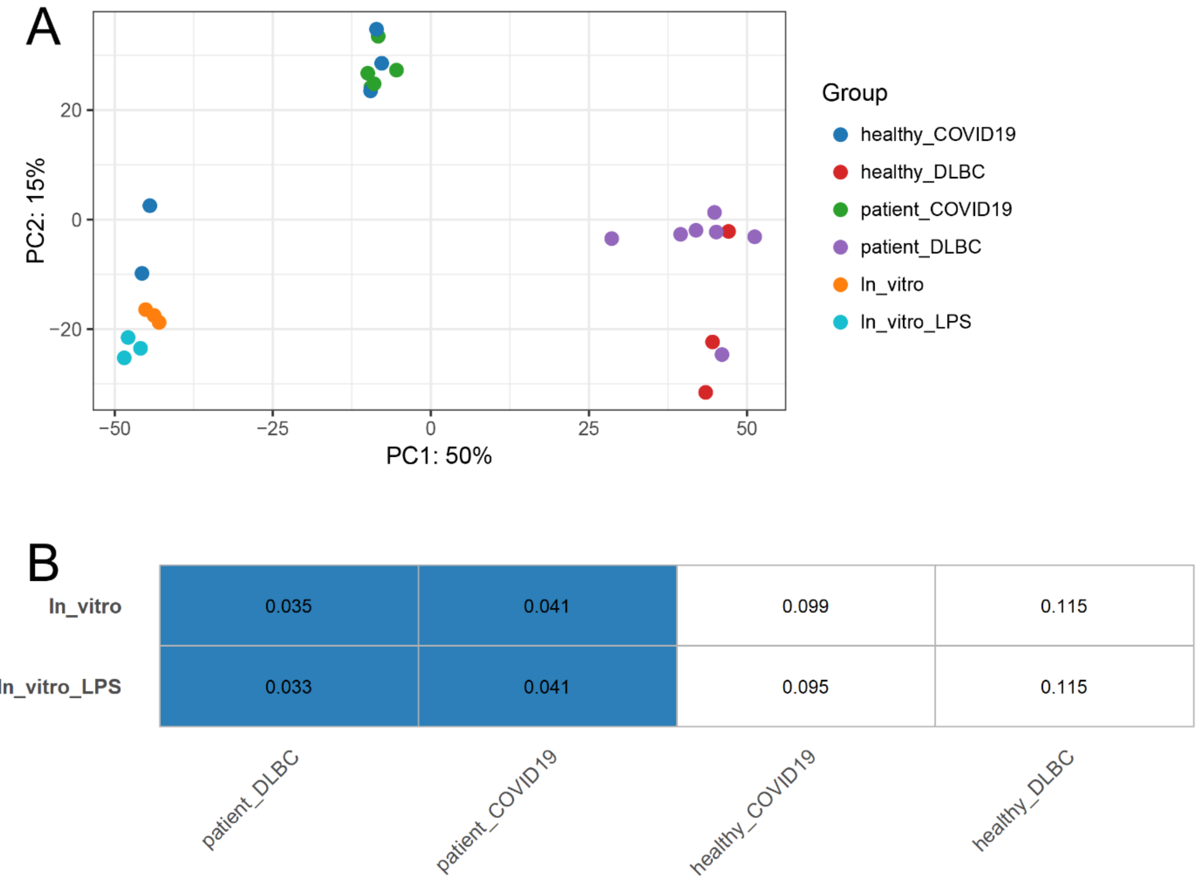

Supplementary Figure S10. Comparative transcriptomic analysis of *in vitro* generated and *ex vivo* moMDSCs. RNA-seq data from in vitro–generated moMDSCs generated in this study (unstimulated, *In_vitro*, or LPS-stimulated, *In_vitro_LPS*) were integrated with publicly available RNA-seq datasets of ex vivo moMDSCs isolated from peripheral blood of patients (*patient_COVID-19*, *patient_DLBC*) and healthy donors from the same studies (*healthy_COVID-19*, *healthy_DLBC*). **A)** Principal component analysis (PCA) of variance-stabilized gene expression profiles reveals the global relationships between in vitro–generated and ex vivo moMDSC populations. Each point represents an individual sample and is colored according to group identity. The percentage of variance explained by PC1 and PC2 is indicated on the axes. **B)** Pairwise PERMANOVA analysis quantifying transcriptomic differences between groups. Heatmap shows FDR-adjusted p-values (Benjamini–Hochberg correction) derived from pairwise comparisons using 999 permutations. Blue cells denote statistically significant differences between the indicated groups (padj ≤ 0.05).

Supplementary Tables

Supplementary Table S2. List of qPCR primers

Target gene (abbreviation)	Forward 5'-3'	Reverse 5'-3'
**sequestosome-1/p62** (SQSTM1)	GCCAGAGGAACAGATGGAGT	TCCGATTCTGGCATCTGTAG
**beclin-1** (BECN1)	CTGGGACAACAAGTTTGACCAT	GCTCCTCAGAGTTAAACTGGGTT
**microtubule associated protein 1 LC3 β (**MAP1LC3B**)**	TTCAGGTTCACAAAACCCGC	TCTCACACAGCCCGTTTACC
**autophagy and beclin 1 regulator 1** (AMBRA1)	GGTGGGAGGAGAGGGGATAG	CGAGGGGCATGTCATCATTT
**oxoglutarate receptor 1 (**OXGR1**)**	CTCGATGTGCAGTTGTAGCCTG	GAGTTCATCCGAACTGGTGAGG
**superoxide dismutase 1** (SOD1)	ACAAAGATGGTGTGGCCGAT	AACGACTTCCAGCGTTTCCT
**heme oxygenase 1** (HMOX1)	GGGAAGATGCCATAGGCTCC	CTCCCAGGGCCATGAACTTT
**hypoxia inducible factor 1α** (HIF-1α)	GTCTGAGGGGACAGGAGGAT	CTCCTCAGGTGGCTTGTCAG
**autophagy related 5** (atg5)	CACAAGCAACTCTGGATGGGATTG	GCAGCCACAGGACGAAACAG
**glyceraldehyde-3-phosphate dehydrogenase** (GAPDH)	GTGAAGGTCGGAGTCAACG	TGAGGTCAATGAAGGGGTC

Supplementary references

1. Platt MO. Multiplex Cathepsin Zymography to Detect Amounts of Active Cathepsins K, L, S, and V. Methods Mol Biol. 2017;1626:239–52.

2. Hemming BC, Gubler CJ. High-pressure liquid chromatography of α-keto acid 2,4-dinitrophenylhydrazones. Analytical Biochemistry. 1979;92(1):31–40.
